# Molecular basis for dengue virus broad cross-neutralization by humanized monoclonal antibody 513

**DOI:** 10.1038/s41598-018-26800-y

**Published:** 2018-05-31

**Authors:** Yee Hwa Wong, Akshita Kumar, Chong Wai Liew, Kannan Tharakaraman, Kannan Srinivasaraghavan, Ram Sasisekharan, Chandra Verma, Julien Lescar

**Affiliations:** 1 School of Biological Sciences, Nanyang Technological University, 60 Nanyang Drive, Singapore, 637551 Singapore; 2 Department of Biological engineering MIT, Cambridge, United Kingdom; 3 Infectious Diseases Interdisciplinary Research group, Singapore MIT Alliance for Research & Technology, Singapore, Singapore; 4 Bioinformatics Institute, ASTAR, 30 Biopolis Street, #07-01 Matrix, 138671 Singapore, Singapore; 5 Nanyang Institute of Structural Biology, Experimental Medicine Building, 59 Nanyang Drive, Singapore, 636921 Singapore

## Abstract

Dengue is a widespread viral disease with 3.6 billion people at risk worldwide. Humanized monoclonal antibody (mAb) 513, currently undergoing clinical trials in Singapore, targets an epitope on the envelope protein domain III exposed at the surface of the viral particle. This antibody potently neutralizes all four dengue virus serotypes in a humanized mouse model that recapitulates human dengue infection, without signs of antibody-mediated enhancement of the disease. The crystal structure of single-chain variable fragment (scFv) 513 bound to the envelope protein domain III from dengue virus serotype 4 was used as a template to explore the molecular origins of the broader cross-reactivity and increased *in vivo* potency of mAb 513, compared to the parent murine mAb 4E11, using molecular dynamics simulations and network analyses. These two methods are a powerful complement to existing structural and binding data and detail specific interactions that underpin the differential binding of the two antibodies. We found that a Glu at position H55 (Glu^H55^) from the second Complementarity Determining Region of the Heavy chain (CDR-H2) which corresponds to Ala in 4E11, is a major contributor to the enhancement in the interactions of mAb 513 compared to 4E11. Importantly, we also validate the importance of Glu^H55^ using site-directed mutagenesis followed by isothermal titration calorimetry measurements.

## Introduction

Dengue is a major mosquito-borne viral disease, whose prevalence recently expanded beyond the tropical and subtropical regions of the globe, with about 3.6 billion people at risk of contracting the disease^1^. Dengue virus (DENV) infects an estimated 390 million people every year^2^. Most infections with DENV lead to asymptomatic or mild disease^3^. However, 1–5% of the total number of infections provokes severe illnesses that present clinically as Dengue hemorrhagic fever or Dengue shock syndrome, leading to approximately 20,000 to 30,000 deaths per year^4^. A major hurdle in developing a safe vaccine for Dengue has been the presence of four circulating serotypes (DENV1-4) against which sufficient cross-protection must be conferred: incomplete protection against any of the four serotypes can lead to exacerbation of the disease during subsequent infections, via the “antibody dependent enhancement” (ADE) phenomenon^5^. ADE is thought to derive from the presence of weakly neutralizing antibodies in the patient serum that promote infection of Fc receptor-bearing cells like monocytes, leading to amplification of virus production and increased disease severity. An attempt to produce a safe Dengue vaccine by passaging the virus in mice was reported as early as 1945^6^. The Sanofi-Pasteur CYD-TDV tetravalent vaccine, which uses the yellow fever vaccine backbone, is now marketed in several countries^7,8^. However, this vaccine requires three booster injections and confers an uneven protection against the various DENV serotypes, with limited protection against DENV2. Protection conferred by this vaccine appears low in children less than 9 years old and disease worsening was observed in some of the younger vaccinated patients^5^. Moreover, the neutralization titers in the serum from vaccine recipients do not correlate well with protection, suggesting an overall moderate efficacy for the CYD-TDV vaccine^2,8^. Other vaccine candidates are advancing towards late clinical trials^9,10^. Alternative/complementary strategies to prevent and treat DENV severe infections consist in antiviral therapies using small molecules interfering with the replicative functions of DENV non-structural proteins and also therapeutic monoclonal antibodies^11–17^. The murine monoclonal antibody 4E11 emerged as a promising candidate for dengue immunotherapy because it is cross-reactive against all four DENV serotypes^11,18^. However, while 4E11 neutralizes DENV1 and DENV2 rather efficiently with IC50 values of 1.1 nM and 0.85 nM respectively, its reported IC50 values for serotypes 3 and 4 are significantly lower at 54 nM and 100 nM respectively (see Table 1 in ref.^13^)^11,15,18,19^. Elegant X-ray crystallographic structural analyses have defined the binding determinants of 4E11 for the epitopes presented by the four DENV serotypes^15^. The epitope bound by 4E11 is centered on the β-strand “A” of the Ig-like domain III from the E protein (DIII)^20^. For therapeutic use, it was desirable to humanize the murine mAb 4E11 and also to significantly improve the neutralization capacity of this humanized antibody towards DENV3 and DENV4, while retaining high affinity towards DENV1 and DENV2. This was accomplished in two stages: (1) using a purely computational approach, an initial improved version of 4E11 named 4E5A, was engineered by introducing five affinity-enhancing mutations in three complementarity determining regions (CDRs): this subset of mutations (CDR-L1: Arg31Lys; CDR-L2: Asn57Glu, Glu59Gln, Ser60Trp; CDR-H2 Ala55Glu) were selected from a total of 87 single mutants tested and their affinity-enhancing effects were shown to be additive (see Tables S8, S9 and S10 in ref.^17^). 4E5A demonstrated a 15-fold increase in affinity for DENV2 and a 450-fold increase in affinity for DENV4 when compared to 4E11, while maintaining its binding affinity for DENV1 and DENV3^19^. Nonetheless, compared to other serotypes, the resulting affinity of 4E5A for DENV4 was still modest for immunotherapy, with K_d_ = 114.2 nM, as measured by SPR (see Tables 2 and S10 in ref.^17^). (2) In a second stage, antibody 4E5A was then humanized and further modifications carried out in its CDR-H1 region, by introducing a sixth mutation *i.e*. Thr33Val (Thr^H33^, Kabat numbering^21^) and removing an elbow in the framework region preceding CDR-H1, via the deletion of Ser26 (this deletion is hereafter named Ser26Δ). As a result, an optimized human antibody named 513 (Fig. S1) was designed that demonstrates neutralization of DENV1-4, with EC_50_ values of <200 ng/mL for all four serotypes^16^. Importantly, 513 is currently undergoing phase I clinical trials for dengue treatment in Singapore^22^. Interactions between 513 and its epitope on the envelope protein were visualized experimentally and compared with the predicted antibody-antigen complex, by crystallizing the recombinant single-chain variable fragment of 513 (scFv513) bound to DIII-DENV4 (isolate Mexico/1997). Two crystal forms were obtained and their structures were reported at 2.49 Å and 3.27 Å resolution respectively (PDB codes: 5AAM and 5AAW)^16^. Here, we used as a template the refined crystal structure of scFv513 bound to the envelope protein domain III from dengue virus serotype 4 at 2.49 Å resolution. The respective complexes between DIII of all four DENV serotypes and scFv were modeled using available crystal structures (Table S1). We further carried out molecular dynamics (MD) simulations to analyze the detailed network and energetics of the interactions established by antibodies 513 and 4E11 with their epitopes on DIII across the four DENV serotypes. We also analyzed in detail the energetics of interactions of 513 with two DENV4 isolates: Philippines/1956 H241 and New Caledonia/2009. These two DENV4 viral isolates are bound by mAb 513 with significantly lower affinities (118.5 and 8.9 nM respectively) compared to DENV1-2. Overall, this work provides a quantitative understanding of the enhanced potency of the humanized mAb 513 compared to its parent mouse antibodies 4E11 and 4E5A, from which it was derived. Finally, validation of the computational predictions was performed using site-directed mutagenesis targeting the hotspot residue H55 from the CDR2 of mAbs 4E11 and 513, followed by isothermal titration calorimetry measurements.

## Results

### Electrostatic surface variations in the epitopes across the four DENV serotypes

**Figure 1:**
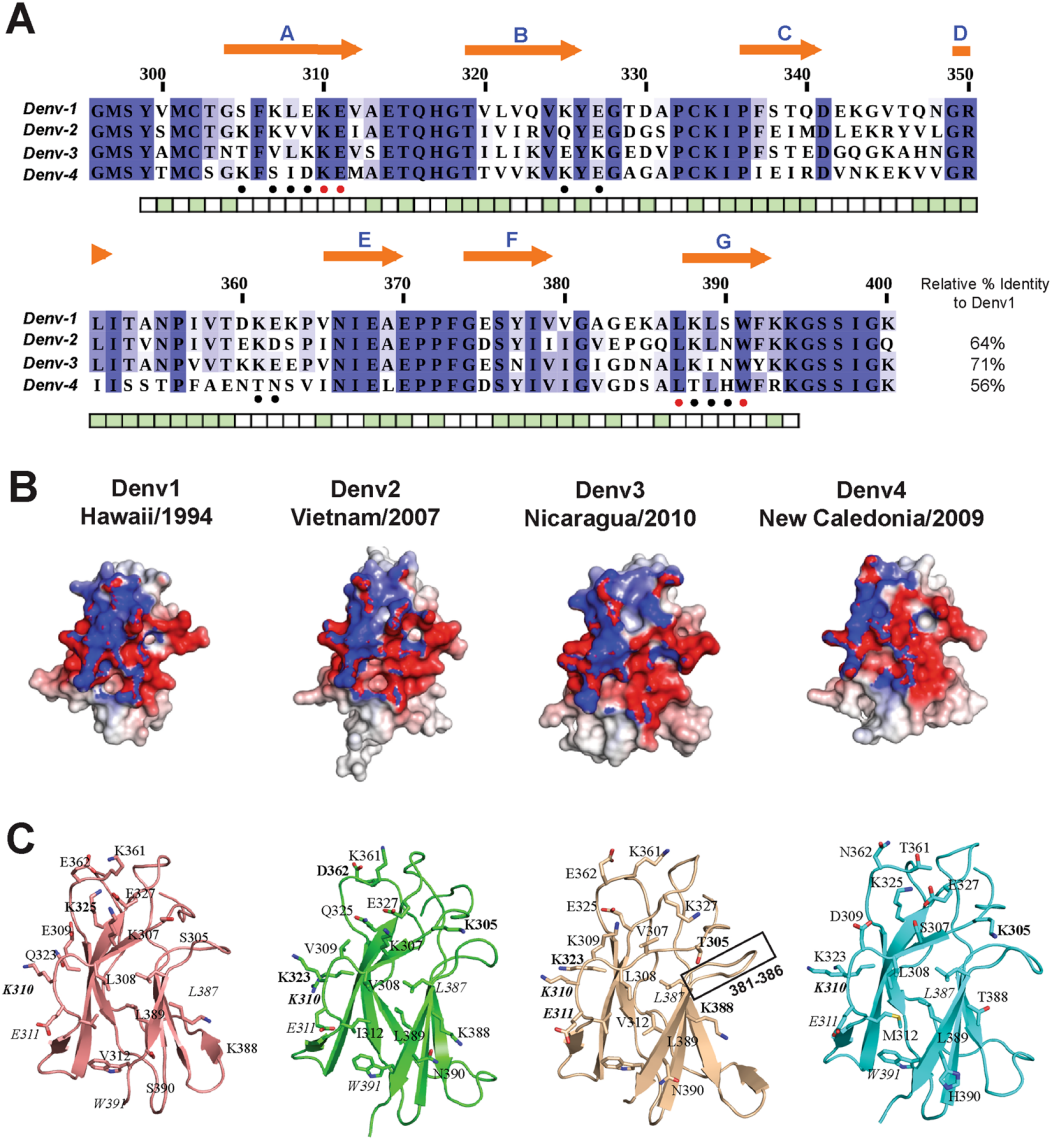
The DENV neutralizing epitope. (**A**) Amino acid sequence alignment of E protein DIII of four DENV serotypes used in this study (listed in Table 2). Residue numbers correspond to the full length DENV E protein. Residues that belong to β-strands (labeled from A–G) are below orange arrows. Residues marked with red and black dots under the alignment, represent conserved and variable epitope residues respectively. Colored blocks depicting the average surface accessibility of residues in white (accessible) to green (buried) are displayed under the alignment. (**B**) and (**C**) Structural comparison of the epitopes from the constructed DIII homology models. The surface charge distribution of the epitopes for the four DENV serotypes are shown in (**B**) The surface electrostatic potential is represented as a color gradient from blue (positive) to red (negative) between ±1 *kT* and was calculated using APBS plugin in PyMOL. Respective panels in C display the epitope residues as sticks. Residues labeled in *italics* are conserved in all four serotypes, while others are mutated across the four serotypes. The region boxed by a rectangle highlights the unique loop orientation adopted by residues 381–386 in DENV3.

An alignment of the amino-acid sequences of DIII domains of DENV1-4 used in this study, spanning amino-acid residues 296-400 from the envelope E protein ectodomain, is shown in Fig. 1A. In agreement with phylogenetic analysis conducted on a large number of DIII sequences from both DENV epidemic and sylvatic strains^23^, DENV1 and DENV3 share the highest amino-acid sequence identity (60–70%) and are both closer to DENV2 than to DENV4. DENV4 apparently diverged earlier with amino-acid sequence identities of only 40–50% with DENV1-3^24^. Thus, as anticipated, the evolution and mutations observed among DENV serotypes correlate directly with the cross-neutralization efficacy of mAbs 4E11, 1A1D-2, 9D12 and 513, all of which bind an epitope centered on the A-strand of DIII^15,18,25–28^. Crystallographic and NMR studies of the flavivirus E protein ectodomain and of isolated DIII domains demonstrated that despite significant sequence variations between the four serotypes, the DIII structure is well conserved: an IgC-like fold with seven β-strands labeled from A to G^15,18,25-28^. The N-terminal region of DIII as well as three strand-connecting loops BC, DE and FG are the most exposed at the virion surface and engage in contacts with various neutralizing antibodies described so far^23^. Moreover, the molecular surface of the viral particle is not a static structure but “breathes”, especially at the physiological temperature of 37 °C, and this dynamic behavior gives antibodies access to epitopes that are partially hidden at lower temperatures^29^. As observed in homology models and experimental crystal structures^14,15^, amino-acid substitutions across the dengue serotypes modulate both the shape of the epitope and its electrostatic properties (Fig. 1B), largely explaining the differences in mAbs 513 and 4E11 binding affinities. Substitutions at residues 361-362 projecting from the DE loop, A-strand (305-312) and G-strand (388–390) are the main contributors to these subtle variations in shape and charge distribution: DENV1, 2 and 3 have Lys at positions 361 and 388, while DENV4 has a polar residue (Thr) (Fig. 1C). Another noticeable alteration of the electrostatic surface is observed at position 307 of the epitope, with the loss of a net positive charge: Lys in DENV1-2 is replaced by Val in DENV3 and by Ser in DENV4. Likewise, position 323 is a positively charged Arg or Lys in DENV2-4 and is replaced by a polar Gln in DENV1. At position 325, a positively charged Lys is present in DENV1 and DENV4, while a Gln is found in DENV2 and a negatively charged Glu in DENV3. The sections below examine in detail the influence of such substitutions on contacts that enhance mAb 513 binding towards the various DENV DIII antigens.

### Analysis of scFv513-DIII-DENV4 contacts from the crystal structure of the complex

**Figure 2:**
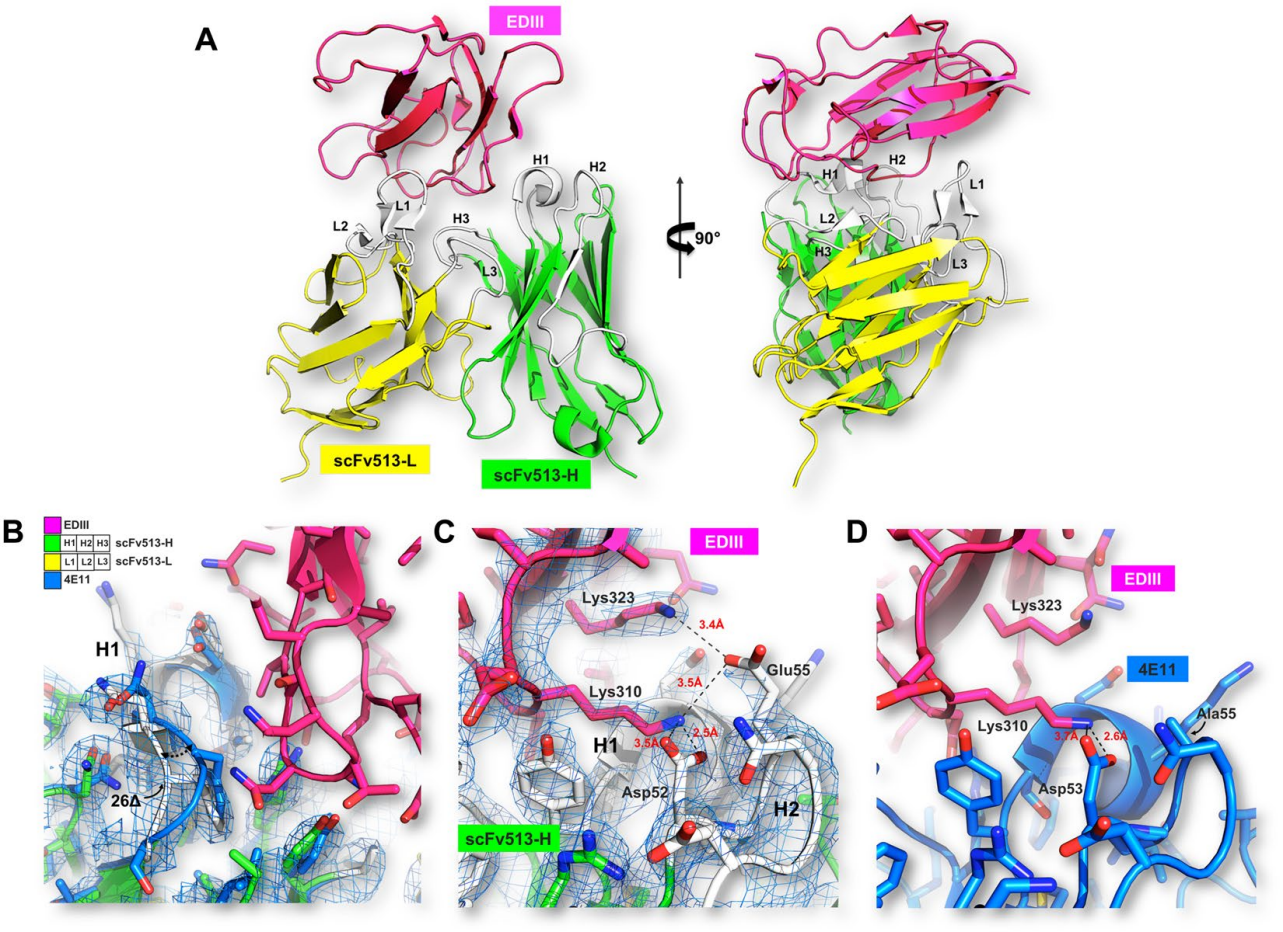
Interactions between scFv513 and DIII from DENV4 derived from crystallography. (**A**) Overall views at two angles separated by 90° of the complex between scFv513 (VH and VL depicted as green and yellow ribbons respectively) and the domain III of the envelope protein from DENV4 (red ribbons). Coordinates were taken from molecule A of PDB code 5AAM. CDRs and N-and C-termini are labeled. (**B**) Details of the interactions and comparison with 4E11: panels (C) and (D) show a key contact region where an additional salt bridge was introduced in scFv513 between Glu55^H2^, (Kabat numbering) and Lys323 (EDIII), which is absent in the complex between scFvE11 and EDIII (panel D). This results from the Ala to Glu substitution in the H2 region of 4E11 (see text). The S26Δ deletion introduced in VH of 513 (white tube) removing the bulge present in 4E11 (blue tube) is displayed in (**B**) and the movement between the two polypeptide chains is shown by an arrow. The electron density map (coefficients 2Fo-Fc at a level of 1σ) is overlaid in panels (B) and (C).

The scFv513 recombinant protein was produced in *E. coli* by linking the variable heavy chain region (VH) of mAb 513 to the variable light chain region (VL). We obtained two crystal structures from the Protein Data Bank (with PDB access codes 5AAM and 5AAW) providing eight views of the complex formed between scFv513 and DIII-DENV4 that was briefly described earlier^16^. An overall view of the complex (Fig. 2A) as well as selected portions of the interactions established between scFv513 and DIII from DENV4 are displayed in Fig. 2, highlighting changes introduced between 513 and 4E11. A close-up view of the Ser26Δ deletion of the elbow in the framework region preceding CDR-H1 is displayed in Fig. 2B. The extra salt bridge between Lys323 (DIII) and Glu55 (CDR-H2) is shown in Fig. 2C and compared with the equivalent region in the 4E11-DIII-DENV4 complex (Fig. 2D**)**. Overall, the scFv513-DIII-DENV4 complex (Fig. 2A) is closely superimposable with the scFv4E11-DIII-DENV4 complex^16^ and a detailed comparison between their atomic contacts and MD studies described below can account for the higher cross-reactivity displayed by 513 (Table 1). In the following sections, we examine the structural dynamics of these complexes and focus on the regions where 513 was modified and establishes contacts with the DIII antigen that differ from those established by the parent 4E11.

**Table 1:** Comparison of intermolecular contacts between DIII DENV4 with mAb513 or 4E11.

**mAb513scFv**	Atom	Distance (Å)	Atom	**DIII DENV4**	Atom	Distance (Å)	Atom	**scFvE11**	**513 vs 4E11**
**CDR-L1**									
Lys31 (163)	NZ	3.0	OE2	Glu311	OE2	2.9	NH1	Arg31 (163)	
Tyr32 (164)	O	2.9	N	His390	N	2.9	O	Tyr32 (164)	
	OH	3.3	N	Met312	N	3.1	OH		
Asn34 (166)	ND2	2.6	O	Thr388	O	2.9	ND2	Asn34 (166)	
**CDR-L2**									
Arg54 (186)	NH2	2.6	O	Phe306	O	3.0	NH2	Arg54 (186)	
Glu57 (189)	OE1	2.8	NZ	Lys305	NZ	3.0	OD1	Asn57 (189)	**sb vs hb**
Gln59 (191)	NE2	3.2	OE1	Glu327	OE1	4.0	OE1	Glu59 (191)	**Hb vs elec repulsion**
Trp60 (192)	CD1	3.4	O	Glu327				Ser60 (192)	
**CDR-H1**									
Lys31 (30)	O	2.8	NZ	Lys310	NZ	2.6	O	Lys31 (30)	
Asp32 (31)	OD1	3.5	NZ	Lys323				Asp32 (31)	
Val33 (32)								Thr33 (32)	
Tyr34 (33)	OH	3.2	N	Glu311	N	3.1	OH	Tyr34 (33)	
**CDR-H2**									
Asp53 (52)	OD1	2.9	NZ	Lys310	NZ	2.6	OD1	Asp53 (52)	
Glu55 (54)	OE2	3.1	NZ	Lys323				Ala55 (54)	**Extra sb**
**CDR-H3**									
Arg99 (98)	NH2	3.1	OD2	Asp309	OD1	3.0	NH2	Arg99 (98)	
					NE	3.2	OD2		
	NH2	3.4	O	Asn362	O	2.7	NH1		
Glu102 (101)	OE2	3.3	N	Ile308	N	3.0	OE2	Glu102 (101)	
Tyr106 (105)	OH	3.2	OD1	Asn362	OD1	2.7	OH	Tyr106 (105)	

Only hydrogen bonds (hb) and salt bridges (sb) with distances < 3.5 Å are shown.

Numbering is according to Kabat convention. The PDB structure numbering is indicated in parenthesis when different from Kabat convention. The substitutions introduced in 513 compared to 4E11 are in bold.

The additional salt bridges (sb) unique to the scFv513-DIII-DENV4 are indicated in bold.

### Overall structure of the complexes derived from MD simulations

**Figure 3:**
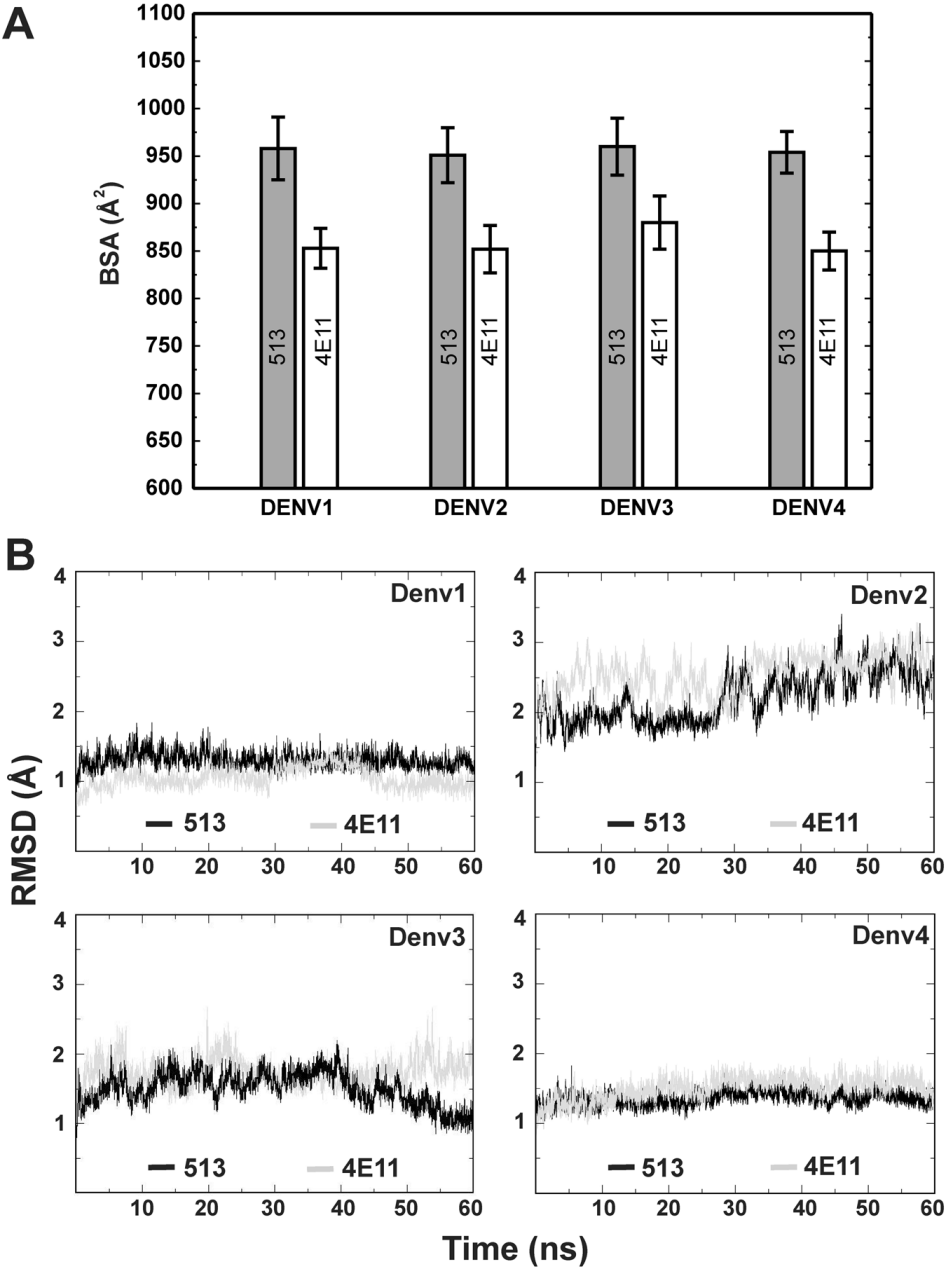
Comparison of 513 and 4E11 complexes with the four DENV serotypes. (**A**) Binding Surface Area (BSA) for the four DENV serotypes DIII in complex with 513 and 4E11. (**B**) RMSD shown for the respective DENV serotypes bound to mAb 513 or 4E11 over a 50 ns MD trajectory.

To reveal the structural details underlying neutralization efficacy and binding variations between 513 and 4E11, MD simulations on atomic models of each scFv fragment bound to DIII from the various serotypes were used. The three complexes between 513 and DIII proteins from DENV1-3 were modeled using the crystal structure of scFv513 bound to DIII from DENV4 as template (PDB code 5AAM). Cartesian coordinates from the experimental crystal structures of 4E11 bound to DIII from the four DENV serotypes were obtained from the PDB (see Table S1)^15^. Each of these eight 3D models was optimized and refined by energy minimization and subjected to MD simulations (see Methods). Overall, the resulting minimized complexes between DIII from each serotype with either 513 and 4E11 can be superimposed with root mean square deviations (RMSD) ranging from 0.7 to 1.0 Å. For DENV serotypes 1 and 4, a larger accessible surface area of DIII becomes buried (depicted as the buried surface area or BSA) upon its interaction with 513 compared to 4E11 (Fig. 3A). In contrast, BSA values are comparable for serotypes 2 and 3 for both mAbs. For both 513 and 4E11, a smaller accessible surface area becomes buried upon interaction with DIII of DENV3 in comparison to other serotypes. This is a consequence of the unique orientation adopted by residues 381-386 in DIII of DENV3 that differs from the other serotypes (Fig. 1C). As a result, this bulging loop restricts the engagement of the antibody in the complex with DIII from DENV3. Per-residue BSA calculations for 513 vs 4E11 (bound to each DENV serotype) indicate that CDR residues are more buried in the case of 513 than 4E11 (Fig. S2). For CDRs of the heavy chain, major differences are observed at residues 25-37 (H1), 50-55 (H2) and 99-108 (H3) and at residues 30-37 (L1) and 52-62 (L2) of the light chain (Fig. S2). Overall, the structures of the DIII from all four DENV serotypes in complex with their respective antibodies are stable during the MD simulations (Fig. 3B) compared to their free states (Fig. S3). Next, we examined the conformations derived from the MD simulations to provide a mechanism for the observed enhanced binding of 513 to DENV4 compared to 4E11.

### 513 shows strong binding to DIII from DENV1-3 and enhanced binding to DENV4

**Figure 4:**
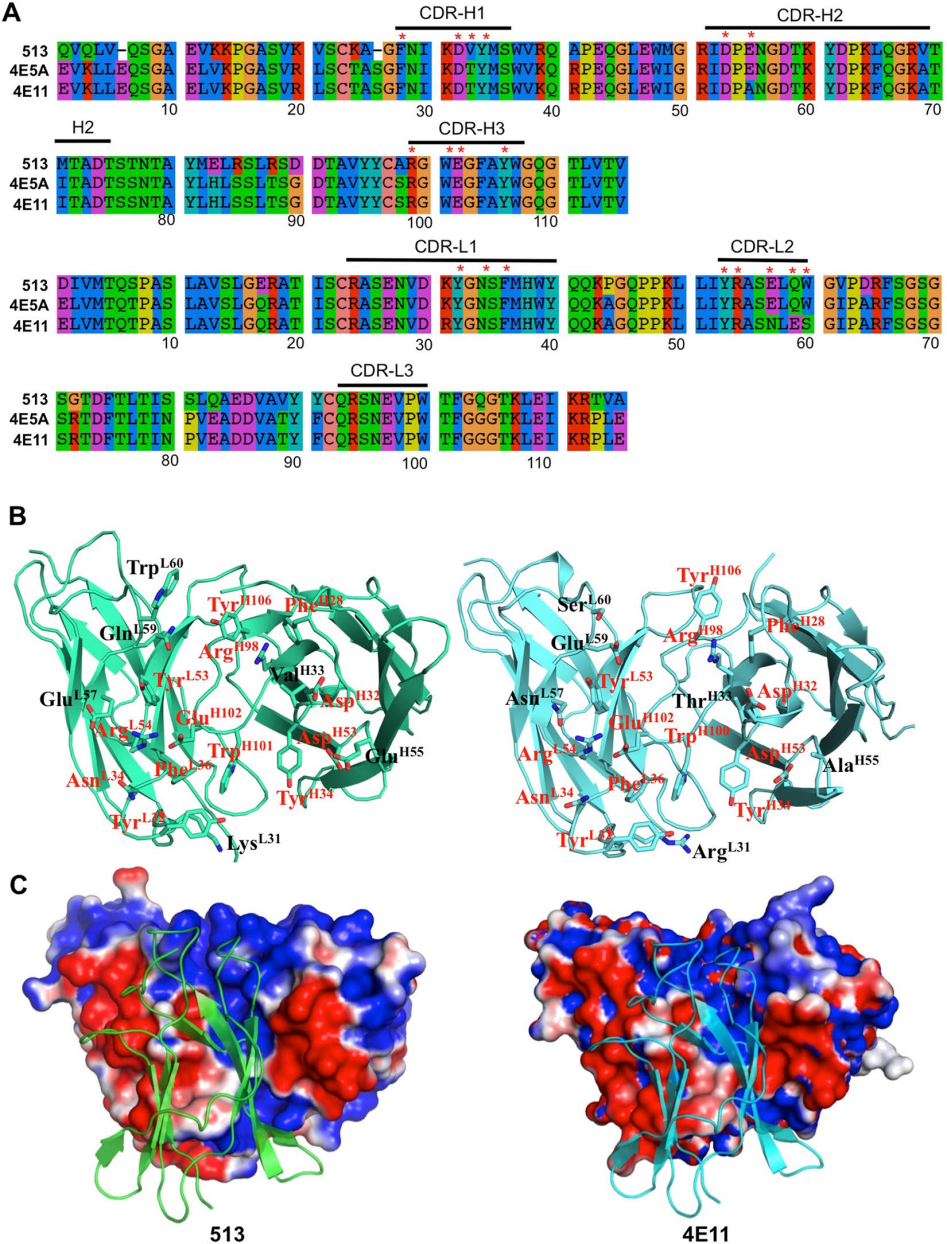
Overview of the 513 and 4E11 paratopes. (**A**) Amino acid sequence alignment of the variable regions from mAb 513, 4E5A and 4E11 (**B**) Side by side view of the two paratopes seen “from the antigen” for mAb513 (left) and mAb4E11 (right). Interface residues are shown as sticks. Conserved and mutated residues are labeled in red and black font respectively. (**C**) Electrostatic surface representation of the paratopes with the DIII protein antigen shown as a green (mAb 513) or blue ribbon (mAb 4E11). The surface electrostatic potential is represented as a color gradient from blue (positively charged) to red (negatively charged) between ±1 *kT* and was calculated using the APBS plugin in the program PyMOL

The binding energy of each mAb to DIII from DENV1-4, was calculated using MM–PBSA (see Methods) averaged over the conformations sampled during the last 30 ns of the equilibrated MD trajectories (Table 2**)**. Overall the binding of 513 is favored over 4E11 in all cases, especially for DENV4 (Table 2). In the case of DENV1-3, the favorable binding energy derives from gains in electrostatics that are offset by desolvation penalties. Side-chains projecting from β-strands A (residues 305–312) and G (387–391) predominantly define the epitope of all four DENV serotypes. The core of the antigen-antibody interface is composed of hydrophobic residues at positions 308, 312, 387, and 389 as well as residues that undergo only conserved substitutions that maintain their aliphatic character and also the overall shape of the epitope. The formation of conserved contacts between both antibodies and the DIII proteins of all serotypes largely explains the cross-reactivity of 513 and 4E11. In this respect, CDR residues Glu^H102^, Tyr^L32^, Gln^L59^ and Trp^L60^ that interact with main-chain atoms from DIII residues located at the center of the interface allow 513 to bind to all DENV serotypes (Fig. 4A,B). While residues Glu^H102^ and Tyr^L32^ are conserved, Gln^L59^ and Trp^L60^ are two of the six affinity-enhancing mutations introduced in 513. Moreover, these residues significantly contribute to the total interaction free energies as seen in the next section. Based on the decomposition of the energy terms, it is clear that the increased affinity of 513 for DIII from DENV4 originates from an improved electrostatic stabilization (Table 2). This is in agreement with the extra salt bridges observed in the crystallographic structure (Table 1). Several substitutions accounting for the observed variations between 513 and 4E11 in binding to DIII from serotype 4 are found at positions 307, 312, 329, 361, 364, 388 and 390 of the epitope (Fig. 1). Residue His390 from DENV4 projects from the G-strand of DENV4 and its backbone atoms form energetically favorable H-bonds with Tyr^L32^. This contact is maintained in other DENV serotypes which have shorter aliphatic side chains at this position: Ser in DENV1 and Asn in DENV2 and DENV3. Another serotype specific contact for DENV4 is observed in the B-strand, where the carboxylic side chain of Glu327 H-bonds the side chain of Gln^L59^ and Trp^L60^ of 513 (Glu^L59^ and Ser^L60^ in 4E11). Due to conformational variability in this region, this contact is absent in other DENV serotypes.

**Table 2:** The various MM-PBSA terms for the binding of DENV serotypes to 513 and 4E11.

Contribution	DENV1		DENV2		DENV3		DENV4(NC)		DENV4(P)	
	**513**	**4E11**	**513**	**4E11**	**513**	**4E11**	**513**	**4E11**	**513**	**4E11**
^*^ΔE_ele_	−388	−436	−599	−515	−375	−466	−488	−375	−495	−356
	(50)	(31)	(40)	(31)	(46)	(35)	(36)	(33)	(45)	(35)
^**^ΔE_vdW_	−120-	−101	−93	−110	−100	−74	−102	−99	−95	−78
	(6)	(6)	(6)	(8)	(7)	(6)	(8)	(5)	(6)	(5)
^***^ΔG_EPB_	428	464	613	551	420	483	519	408	519	403
	(41)	(32)	(36)	(46)	(40)	(31)	(33)	(29)	(37)	(37)
^$^ΔG_Cavity_	−13	−11	−12	−13	−12	−11	−12	−11	−12	−11
	(0.4)	(0.3)	(0.3)	(0.4)	(0.5)	(0.3)	(0.6)	(0.3)	(0.3)	(0.5)
^$$^ΔG_gas_	−509	−538	−692	−625	−408	−540	−590	−473	−590	−434
	(47)	(31)	(39)	(33)	(40)	(35)	(37)	(32)	(44)	(33)
^#^ΔG_sol_	415	452	601	537	475	472	506	396	506	392
	(39)	(35)	(36)	(32)	(46)	(31)	(33)	(28)	(37)	(33)
ˆΔG_bind_	−94	−86	−90	−87	−67	−67	−83	−76	−83	−42
	(8)	(7)	(4)	(3)	(11)	(11)	(7)	(9)	(11)	(9)

All units are given in kcal/mol.

^*^ΔE_ele_: electrostatic energy.

^**^ΔE_vdW_: van der Waals energy.

^***^ΔG_EPB_: the electrostatic contribution to solvation free energy.

^$^ΔG_Cavity_: nonpolar contribution to the solvation free energy.

^$$^ΔG_gas_: gas-phase energy.

^#^ΔG_sol_: solvation energy.

ˆΔG_bind_: estimated binding free energy.

Standard deviations of corresponding values are given in parentheses.

DENV4 (NC) corresponds to New Caledonia strain; DENV (P) Corresponds to Philippines strain.

### Energetic impact of substitutions in 513 compared to 4E11

**Figure 5:**
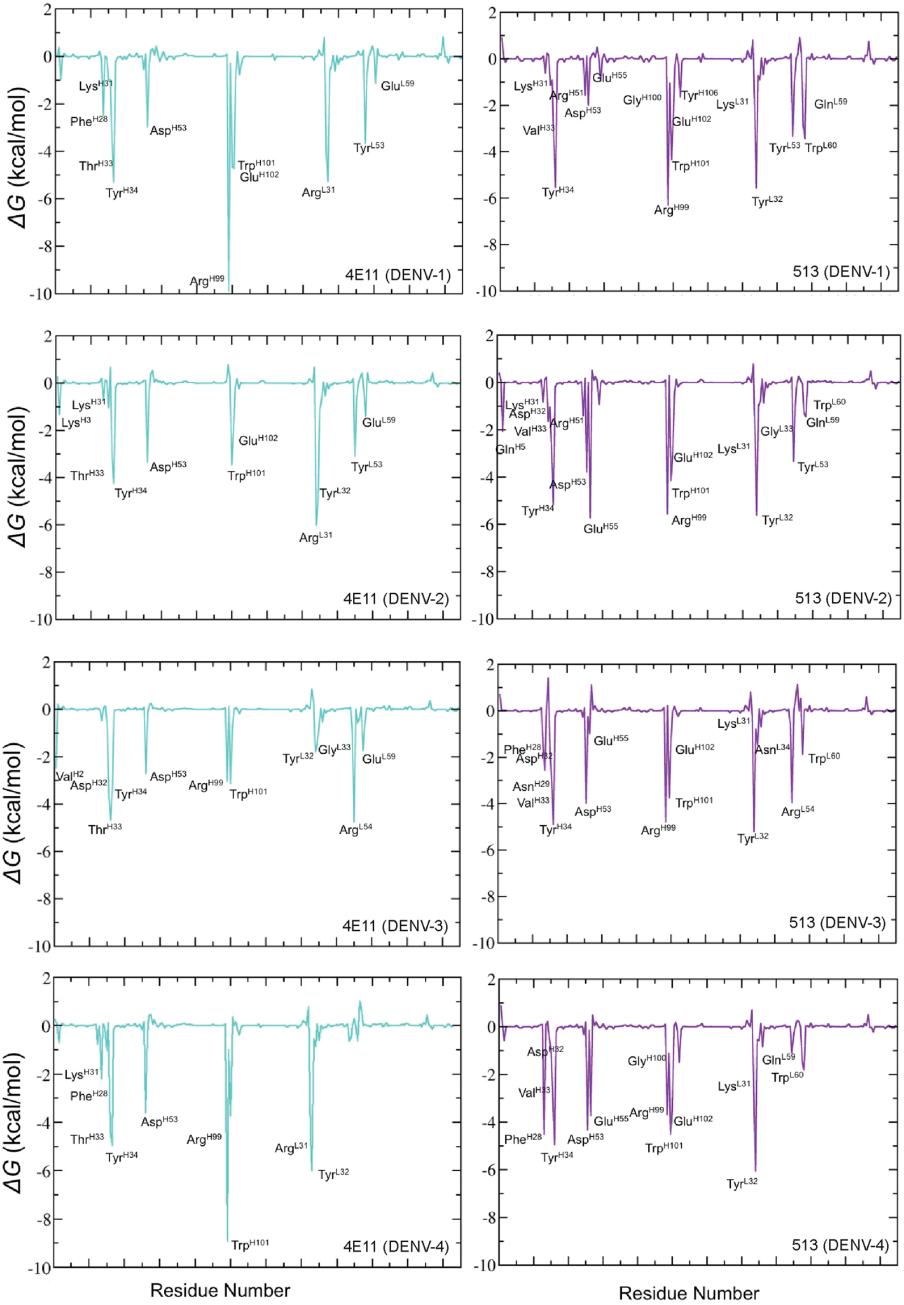
Analysis of the energetics of DIII-antibody interactions I. Each plot depicts the free energy contributions of residues from the mAb 4E11 (left panels) and 513 (right panels) combining sites, when bound to DIII from each of the four DENV serotypes.

**Figure 6:**
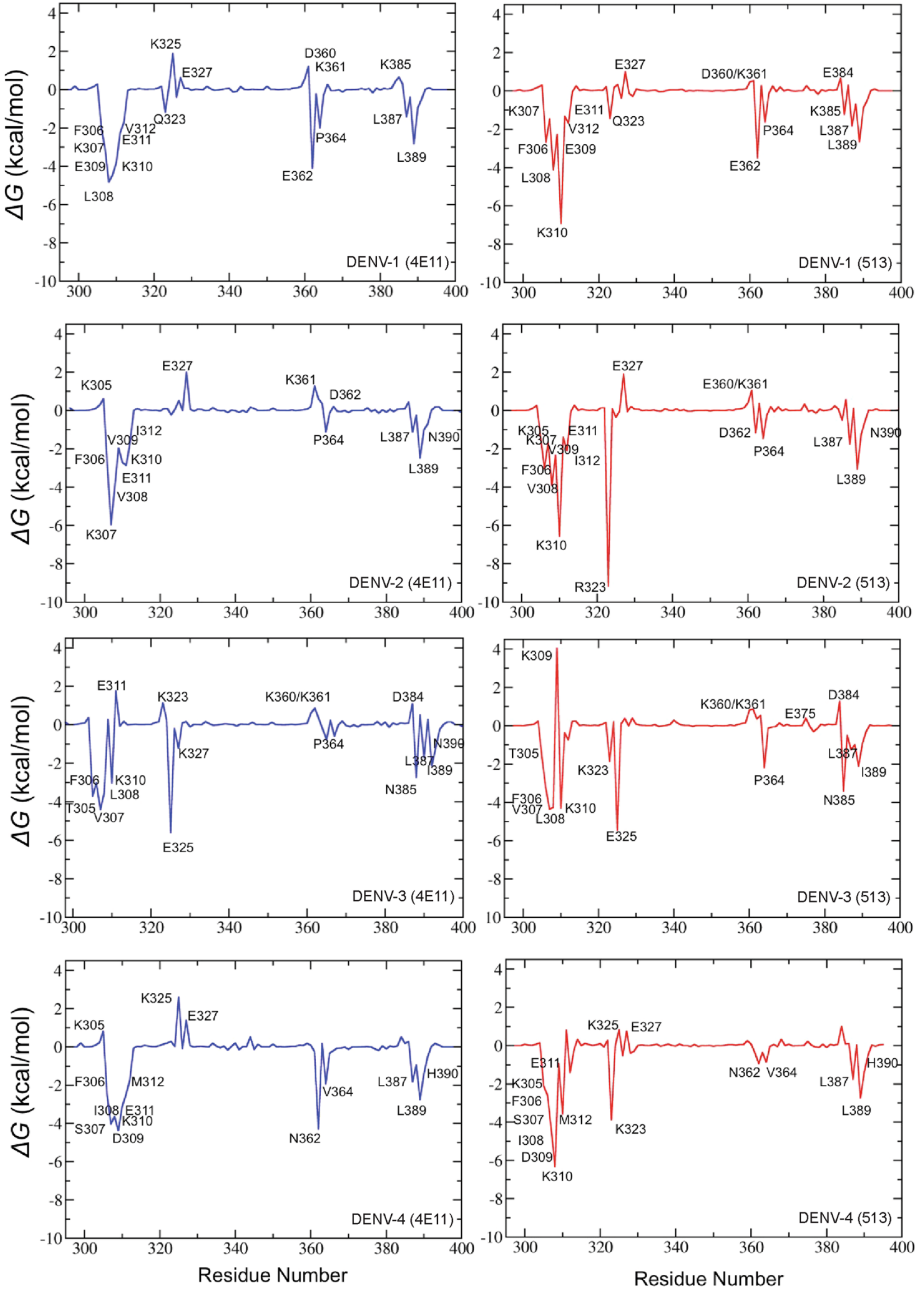
Analysis of the energetics of DIII-antibody interaction II: Each plot depicts the free energy contributions per DIII residues from each DENV serotype, in complex with 4E11(blue, left panels) or 513 (red, right panels).

**Figure 7:**
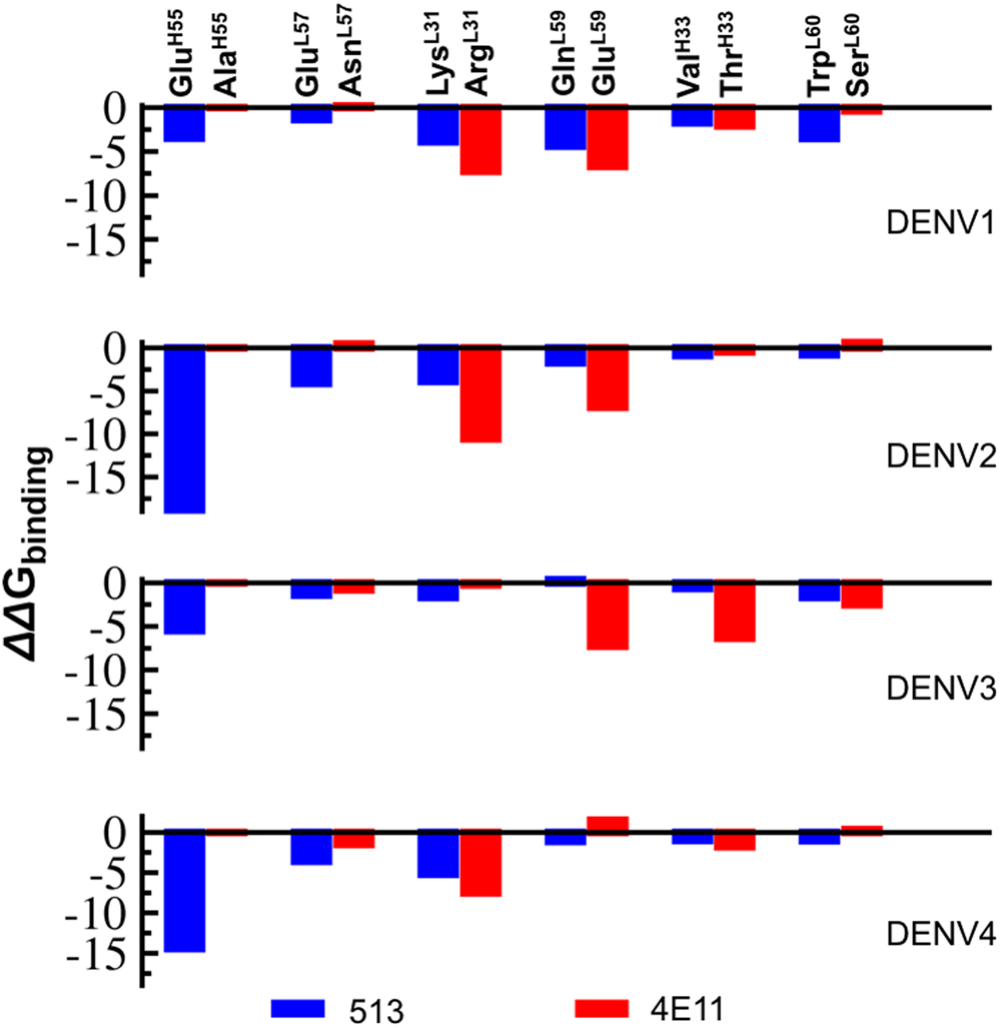
Computational mutagenesis performed on six key affinity-enhancing substitutions introduced in 4E11 to generate 513, when bound to the four DENV serotypes. Each residue in the respective complex was mutated to Alanine and the corresponding ΔG value was calculated. Each ΔG value was subtracted from the ΔG value when the original residue was present and the difference is plotted as ΔΔG.

mAb 513 was engineered from 4E11 by the introduction of six affinity-enhancing mutations: Thr^H33^Val (CDR-H1), Ala^H55^Glu (CDR-H2) of VH, Arg^L31^Lys (CDR-L1), Asn^L57^Glu, Glu^L59^Gln and Ser^L60^Trp (CDR-L2) of VL and Ser26Δ in VH that provided higher surface complementarity (Fig. 2). Remarkably, these substitutions guided by computational modelling enhanced 513 affinity for DIII-DENV4 in the experiments, without affecting the affinity to other serotypes^15^. The impact of these substitutions can be seen in Fig. 5, which shows the free energy contributions of antibodies residues with each of the four DENV serotypes. From the antigen side, a similar energetic analysis is shown in Fig. 6. In addition, these six affinity-enhancing mutations were also subjected to “computational alanine scanning” to evaluate individual side-chain contributions to the overall binding energies. When these ‘hot spot’ residues were mutated to Alanine in either 513 or 4E11, we observed an overall destabilization of the respective antigen-antibody interface, as measured by the *ΔΔG* binding energy (Fig. 7). Across all serotypes, residues Glu^H55^, Lys^L31^ and Glu^L57^ contribute favorably to 513 binding. Using this “computational mutation scanning analysis”, Glu^H55^ from CDR-H2 in 513 appears to be a major contributor towards the enhancement of the binding energy, as analyzed below in greater detail.

### The Ala55Glu mutation in CDR-H2 dramatically improves the affinity of 513 for DENV4

In the crystal structure, the carboxylic side-chain of Glu^H55^ forms a salt-bridge with the side-chain of Lys323 (Fig. 2C), a basic residue conserved in serotypes 3 and 4 and substituted by Arg in DENV2 and Gln in DENV1. These three side-chains from the antigen provide comparable favorable electrostatic interactions with Glu^H55^, with Gln unsurprisingly being the least favorable (Fig. 6). These interactions were recapitulated in the MD simulations of the respective DENV serotypes in complex with either 4E11 or 513. Upon binding, no major structural changes were observed in the chi1, chi2 and chi3 distribution plot of Glu^H55^ (Fig. S4). Contributions of individual residues from the CDRs to the total interaction energy show that Glu^H55^ from 513 contributes significantly (up to −6 kcal/mol) except in the case of DENV1 and DENV3 (Fig. 5). Likewise, residues at position 323 in DENV contribute significantly to the interactions with 513 (Fig. 6). Structural examination of DIII from the four serotypes in complex with 513 reveals that stable salt bridges and hydrogen bonds mediate the interactions between side chains of positively charged (Lys/Arg) residues at position 323 in DENV 2, 3 and 4 with Glu^H55^ and Asp^H32^ (with over 90% occupancy). In the case of DENV1, the carbonyl atom of Asp^H32^ forms a stable H-bond with the Gln323 side chain, with 85% occupancy in both 513 and 4E11. The introduction of a negative charge at Glu^H55^ in CDR-H2 in the 513 paratope is also likely to favor binding, because of the proximity of Lys310 in DENV1-4 (Figs 1A and 2C). Lys310 has been classified as a conserved epitope residue for 4E11 epitopes^15,18,25–28^. It belongs to a bulging loop with conserved shape and charge complementarity across all serotypes (Fig. 1B,C). In the crystal structures, the key interactions between the side chains of Asp^H31^ (conserved in both 4E11 and 513) and Lys310 of DIII in all serotypes is strictly conserved in the complexes for each serotype^15,18,25–28^. The energetics of interactions computed from the MD simulations show that Lys310 contributes an additional 1–4 kcal/mol towards complex formation for 513 compared to 4E11 (Fig. 5A). Interestingly, MD simulations suggest that this is a consequence of the formation of a salt bridge between the side chains of Glu^H55^ and Lys310, with over 75% occupancy, across all four serotypes. This interaction is obviously missing in 4E11 because of the absence of charge as well as shape complementarity due to the presence of Ala^H55^ at this position. However, in the aforementioned complexes, Lys310 forms a stable H-bond interaction (over 80% occupancy) with Asp^H53^ in all DENV serotypes. This interaction is also observed in the case of 513 with similar occupancy.

### Experimental validation of residue H55 as a key determinant of the binding affinity

In order to validate experimentally the crucial role of residue H55 for the interaction that was predicted computationally (Fig. 5) and also by a simple visual inspection of the structures (Fig. 2), we targeted this residue from the paratope in both mAb 513 and the parent mAb 4E11. Hence, we introduced the mutation GluH55Ala in scFv513 and conversely the AlaH55Glu mutation in scFv4E11. Using ITC, we then experimentally measured the dissociation constants Kd of these two single mutant antibodies for DIII from DENV2 and compared them with the wild-type (WT) scFv proteins. The reaction parameters (equilibrium dissociation constant K_d_, enthalphy variation ΔH and stoichiometry N) are summarized in Table 3 for each mutant and WT. As shown in Fig. S5, using ITC, we determined that the WT scFv513 bound to DENV2 DIII with a K_d_ of 1.64 ± 7.44 nM while the scFv513 bearing the single mutation GluH55Ala suffered a ~17.5 fold loss in affinity with a K_d_ of 28.7 ± 19.4 nM, close to the WT scFv4E11 which has Ala at position H55 (K_d_ = 30.7 ± 20.6 nM). Conversely, scFv4E11 bearing the AlaH55Glu mutation showed an improved K_d_ of 12.8 ± 10.7 nM (Fig. S5). Taken together, this experimentally validates position H55 at the antibody paratope as a hot spot for the binding energetics and shows that the other five modifications introduce in 4E11 to obtain 513 also contribute favorably to the binding energy.

**Table 3:** Experimental binding parameters derived from ITC for the binding of 513 and 4E11 and single mutants at position H55 of CDR2 of the heavy chain to DIII of DENV2. Experimental details are given in the text and in Fig. S5.

scFv	K_d_ (nM)	ΔH (kcal/mol)	N (sites)
scFv513	1.64	−4.28	1.01
scFv513 GluH55Ala	28.7	−1.78	0.99
scFv4E11	30.7	−2.09	0.96
scFv4E11 AlaH55Glu	12.8	−1.89	1.04

### Substitutions in CDR-L2

Interestingly, despite the well-documented dominance of the VH region for determining antigen recognition and affinity, we found that substitutions in the CDR-L1 and CDR-L2 of 4E11 played an important role in affinity improvement displayed by 513. The Asn57Glu mutation (CDR-L2) provides a higher charge complementarity for 513 compared to 4E11. In the crystal structure, Glu^L57^ (Asn in 4E11) forms a salt bridge with Lys305 in DENV4, a polymorphic residue across the various DENV serotypes. While serotypes 2 and 4 have Lys at this position, serotypes 1 and 3 have small polar side chains: Ser and Thr respectively. The effect of this mutation can be clearly observed as a significant change in the energy contribution (from positive to negative) of Lys305 in serotypes 2 and 4 (Fig. 6). This originates from a strong and long-lived (over 90% occupancy) H-bond between Glu^L57^ and the conserved Lys305 in serotypes 2 and 4 (Table S2). This salt bridge contributes to the improved binding of 513 with serotypes 2 and 4. In contrast, Ser305 in serotype 1 is unable to engage in any H-bond interaction and hence makes no contribution (Fig. 6). On the other hand, the side-chain oxygen atom of Thr305 (DENV3) H-bonds with Arg^L54^ (NH1), however with a moderate strength (distance 3 Å) and lifetime (70% occupancy) in both 4E11 and 513 complexes (Table S2). Another substitution in CDR-L2 that significantly contributes towards binding energy is Glu59Gln (4E11 to 513). The effect of this mutation was examined in the MD simulations, where a stable H-bond is observed between the side chains of Gln^L59^ and Lys307 in serotypes 1 and 2 with 513 (Table S2). The Lys307 side-chain (DENV1 and 2) also forms an additional H-bond with the backbone atoms of Glu^H102^, when in complex with 4E11 and 513. Position 307 is another polymorphic site with a positively charged Lys in serotypes 1 and 2 and Val and Ser in serotypes 3 and 4 respectively. Due to a loss of a positive charge in serotypes 3 and 4 at this position, no favorable/stabilizing interaction is observed in the complex with 4E11. However, the side chain of Ser307 in serotype 4 is stabilized by a strong H-bond (over 95% occupancy) with the Glu^H102^ side chain (Table S2). Another key observation noted in the 513-DIII-DENV4 complex is that Gln^L59^ interacts with Glu327 with more than 90% H-bond occupancy (Table S2, Fig. S6). No such interaction is observed in the complex with 4E11 due to the presence of Glu^L59^ at this position (Fig. S6). Gln^L59^ in the 513-DIII-DENV4 complex contributes ~ −1.5 kcal/mol towards the total binding energy, as opposed to Glu^L59^ in the 4E11-DIII-DENV4-complex, which has an unfavorable total energy contribution of ~ 1 kcal/mol (Fig. 5). The per-residue energy decomposition suggests that Glu^L59^ in DENV4-4E11 causes electrostatic repulsions between negative charge (E_*ele*_ = 14 kcal/mol for Glu), which is removed by mutation to an uncharged Gln^L59^ residue in 513 complex and is therefore electrostatically favorable (E_*ele*_ = −5.5 kcal/mol for Gln). Thus, the Glu59Gln substitution improves the binding of 513 for DENV4.

### Identification of hotspot residues in 513 using orthogonal inter-residue interaction approach

**Figure 8:**
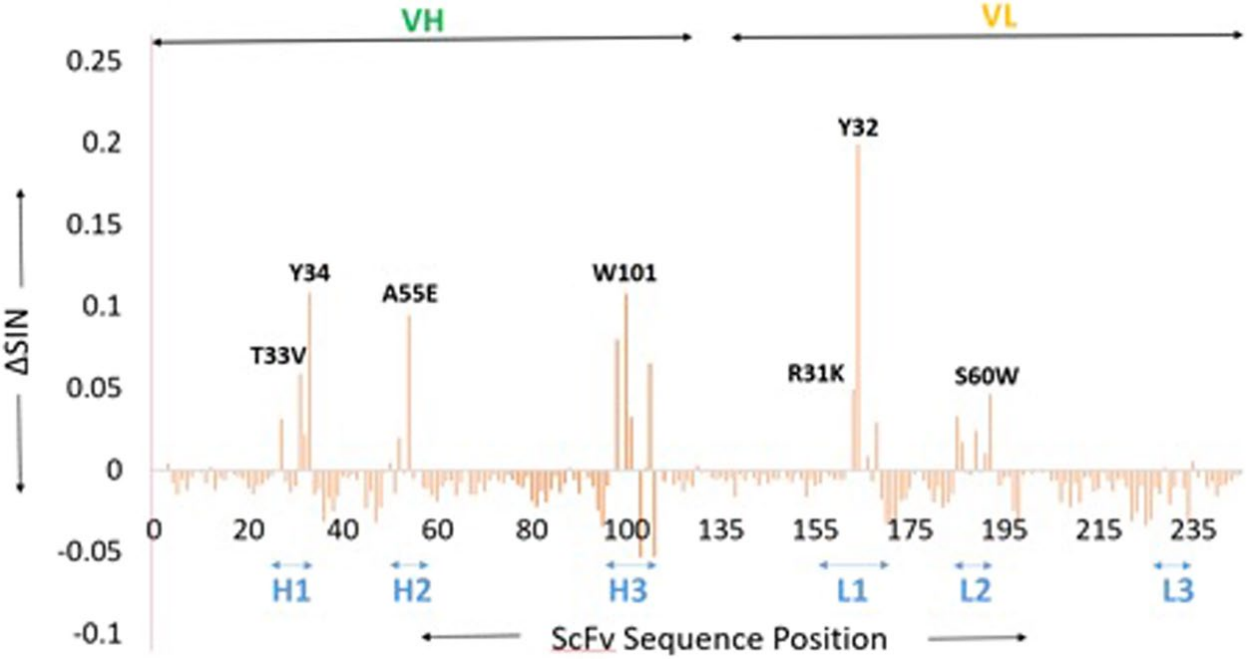
Changes in network scores of ScFv513 plotted over sequence position (PDB: 5AAM numbering followed). The regions corresponding to the heavy and light chains of ScFv513 are indicated above the plot. The locations of the CDR loops are indicated below the X-axis. Residues positions that undergo a large increase in network score are labeled.

Aside from binding energetics and SASA calculations, we employed an orthogonal inter-residue interactions approach^30,31^ to investigate the contributions of ScFv513 residues on DIII binding. This approach enables a quantitative analysis of interactions by computing a numerical network score for each residue based on its interatomic interactions with other residues in its neighborhood^30,31^. The numerical network score for a residue can be correlated to its mutability; the higher the network score, the lower the mutability. Extrapolating from this concept, it is expected that the network scores of paratope residues of an antibody would increase in the antigen-bound state, due to the new pairwise interactions in the epitope-paratope interface. Consequently, residues experiencing a large increase in the network score are likely to be critical hotspots. Next, we computed the differences in the network score of ScFv513 residues in the bound (PDB: 5AAM) versus unbound states. As expected, residue positions undergoing network score changes are restricted to the six CDR loops (Fig. 8). Consistent with the predictions made by the MD simulations, Ala55Glu (CDR-H2) experiences a large increase in the network score upon binding DIII. The network score of affinity enhancing substitutions Thr33Val (CDR-H1), Arg31Lys (CDR-L1) and Ser60Trp (CDR-L2)^19^ also increase (Fig. 8**)**. Unexpectedly, Asn57Glu and Glu59Gln (CDR-L2), which led to ~4-5 fold increase in binding affinity towards DENV4, did not experience network score increase. Residue positions other than the affinity enhancing substitutions that showed large increase in network score (e.g. Tyr^H34^, Trp^H101^, Try^L32^) (Fig. 8) are known hotspot residues, whose modification leads to significant drop in DIII binding^19^.

### Variations in 513 binding to DENV4 Philippines and New-Caledonia isolates

**Figure 9:**
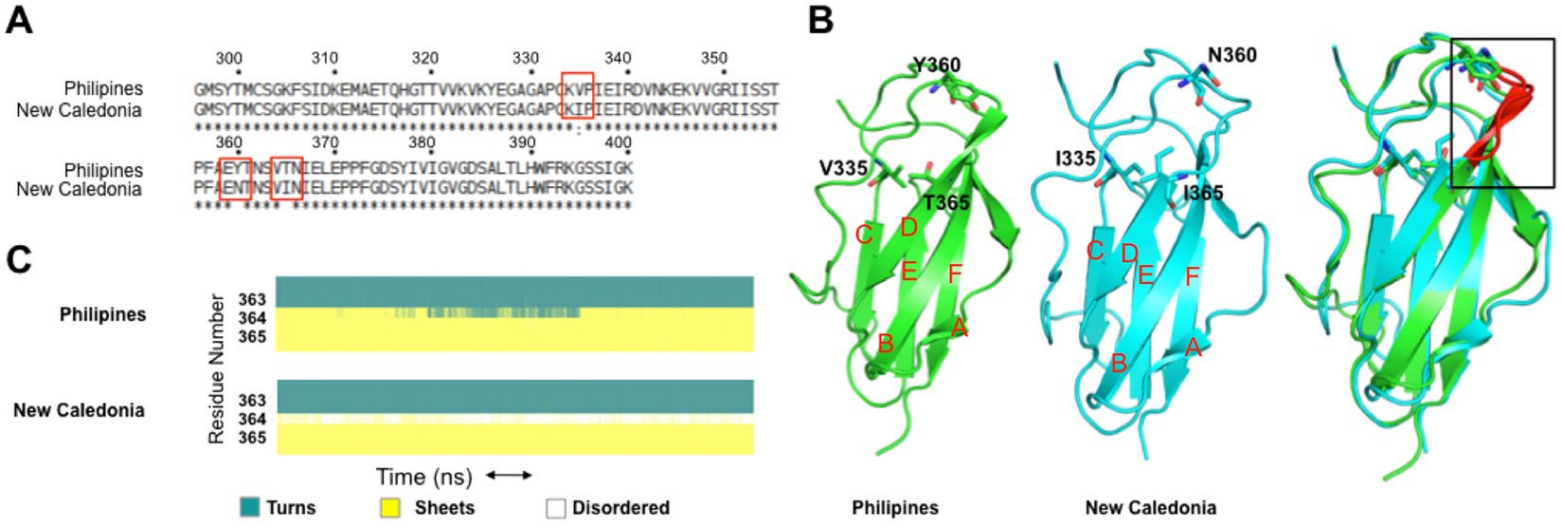
Differences observed in the DENV4 (Philippines vs. New Caledonia) serotype that influence binding of the neutralizing antibody. (**A**) Amino-acid sequence alignment between DENV4 serotypes Philippines and New Caledonia, with the three mutated residues shown in red boxes. (**B**) Variant residues between the two DENV4 strains are shown as ball and sticks on the DIII structure. (**C**) Evolution of secondary structure for residues 363–365 in the respective DENV4 strains during MD simulations plotted using VMD. Residue 364 in New Caledonia adopts a disordered conformation.

**Figure 10:**
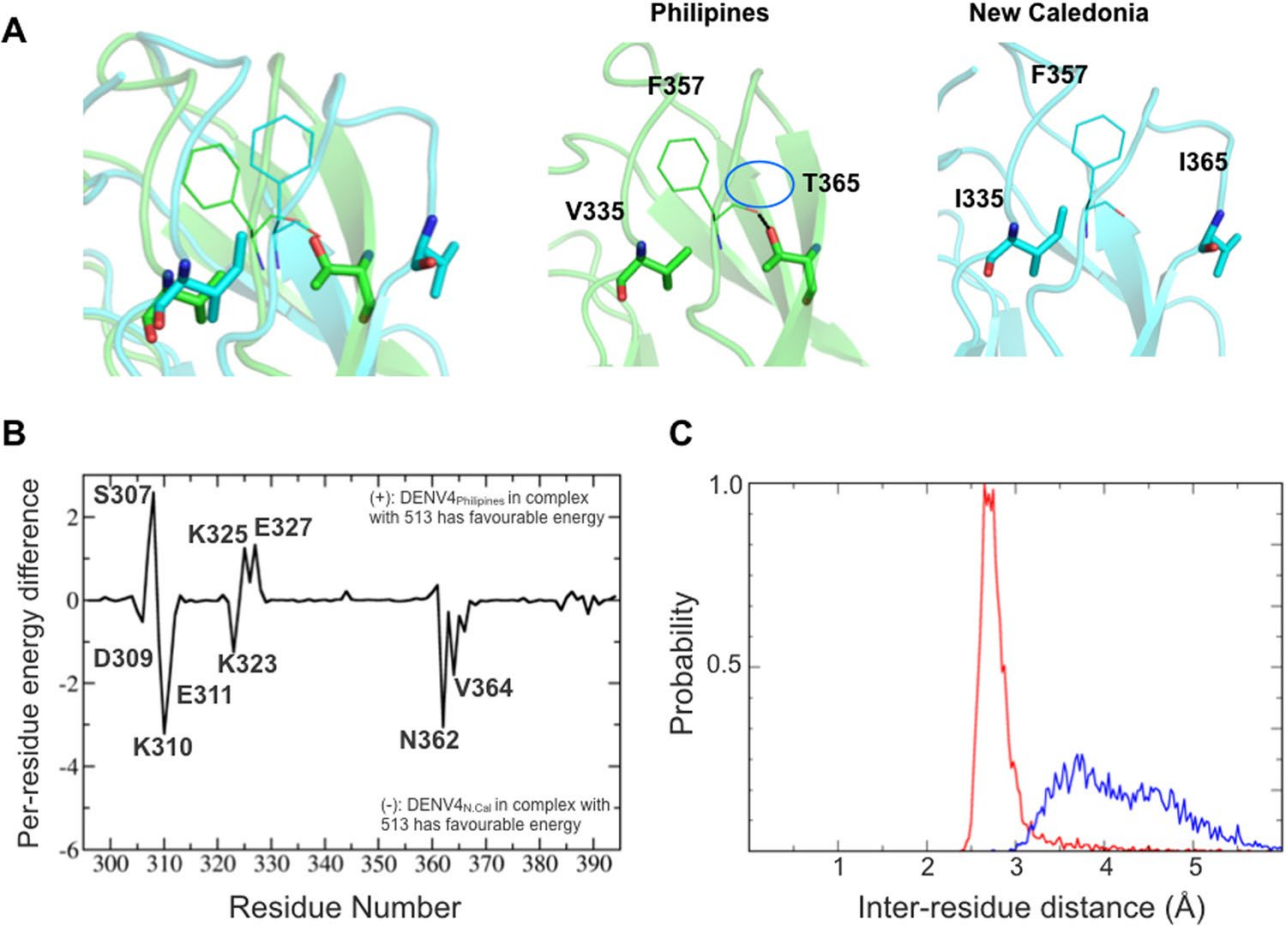
Structural differences within DENV4 serotype between New Caledonia and Philippines isolates (**A**) Superimposed 3D structures of Philippines (green) and New-Caledonia (cyan) bound to 513 (not shown). The reorientation of F357 is shown in the left panel. The middle panel shows a representative snapshot from the trajectory, with the H-bond between the main-chain oxygen of F357 and OG1 of T365. The right panel shows the reorientation of F357 and I365. (**B**) Per-residue energy decomposition difference between DENV-DIII (DENV4 Philippines and New Caledonia) bound to 513; positive values indicate favorable binding energy of the residues from the Philippine strain while negative values indicate favorable binding energy for New Caledonia strain residues. (**C**) Plot of inter-residue distance between F357 and T365 (DENV4 Philippines strain in red) and F357-I365 (New Caledonia DENV4 strain, blue) over the equilibrated trajectories; the plot shows that the strong H-bond between F357:T365 maintains a more compact conformation, with an average bond length of 2.8 Å, while the corresponding conformation is loose in the DENV4 New Caledonia.

**Figure 11:**
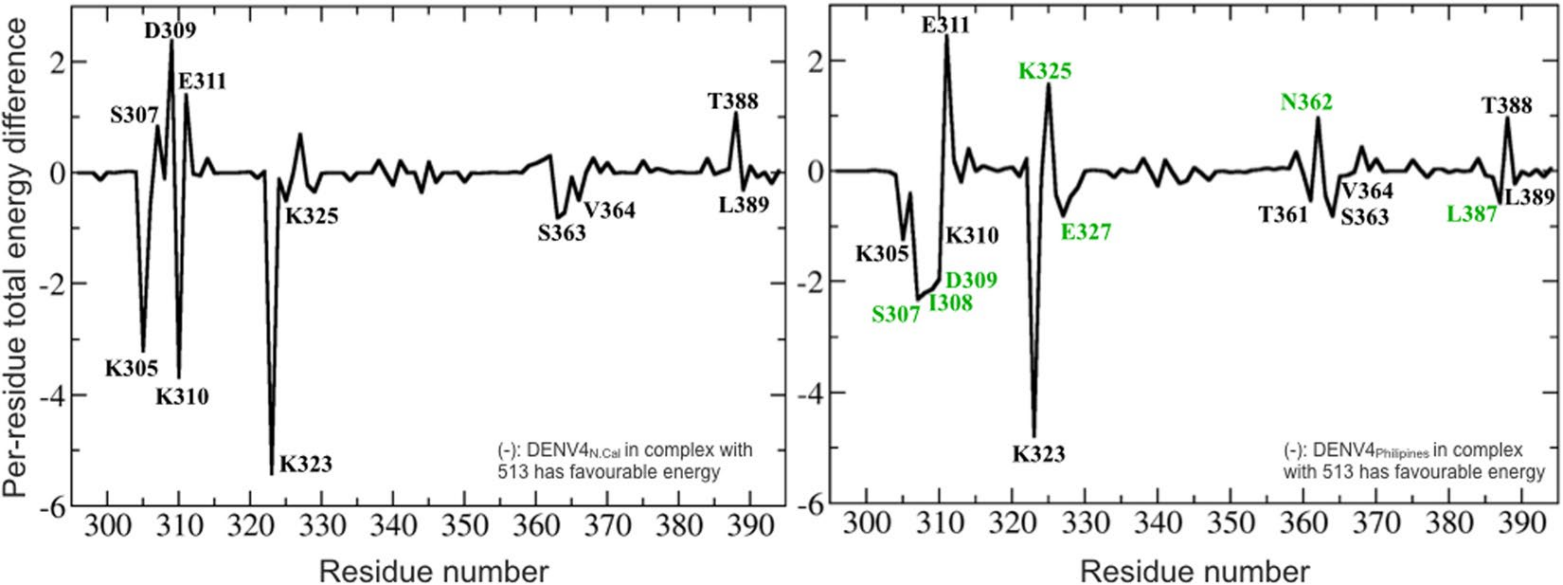
Analysis of energetics of DENV4_New Caledonia_ and DENV4_Philippine_ isolates (right panel). Each plot depicts the free energy contributions of residues from the DENV4_New Caledonia_ (left panels) and DENV4_Philippine_ (right panels), when bound mAb 4E11 and mAb 513. For each isolate, a negative value depicts favorable contribution to energetics when bound to mAb 513, whereas a positive value depicts favorable contribution to energetics when bound to mAb 4E11.

Having defined the origin for the improved binding of 513, we next examined why 513 is more potent than 4E11 against the DENV4_N.Cal_ isolate than against DENV4_Philippines_. Experimental *K*_*d*_ values show a 10-fold difference between these two DENV4 isolates^15^. To characterize these differences in binding affinity of 513 within the DENV4 serotypes observed experimentally, we analyzed the structure and dynamics of the two complexes with either 513 or 4E11. The sequences of DIII from these two DENV4 isolates (New-Caledonia and Philippines) are 97% identical and differ by only three mutations, namely V335I, Y360N, T365I respectively (Fig. 9A). Among these, only residue 365 is near the epitope. The conformations of the two proteins in complex with 513 display an r.m.s.d. of ~1 Å, indicating some differences in antibody positioning: the CDR-L1 is shifted with respect to the end of the A-strand, leading to a loss of charge-charge interactions between Glu311 and Lys^L31^. The AG loop and G strand, that form the core of the epitope, are also affected. Polar contact between Thr388 and Asn^L34^ are also lost. In DENV4_N.Cal_ all three residues 335, 360 and 365 belong to flexible loops, while in DENV4_Philippines_, Thr365 is a part of a β-strand. During the MD simulations, residues 363–365 belong to a disordered region in DENV4_N.Cal_, while in DENV4-_Philippines_ a stable β-strand is observed throughout the trajectory (Fig. 9B,C). Comparison of energy contributions of DIII residues from the two strains bound to 513 shows that residues 362–365 of DENV4_N.Cal_ exhibit favorable binding energies in both electrostatics and van der Waals components (Fig. 10). In the DENV4_Philippines_-513 complex, the Thr365 side chain forms an intra-molecular H-bond with the Phe357 backbone (in the adjacent loop), thus restraining the secondary structure in this region (Fig. 10). However, in the DENV4_N.Cal_-513 complex, Phe357 is re-orientated due to the longer side chain of Ile335 (Val335 in DENV4_Philippines_). Due to this altered conformation, Phe357 no longer interacts with Ile365 (Fig. 10C) making the corresponding regions more dynamic. However, this structural flexibility allows for spatial adjustment (induced fit) for recognition by the antibody. A comparison of the buried surface area (BSA) of DIII DENV4_Philipines_ bound to 513 compared to 4E11 indicates a ≅3-fold increase (*BSA*_4E11:DENV4Philippines_ = 318 Å and *BSA*_513:DENV4Philippines_ = 900 Å). Differences in energy contributions of residues from DENV4_Philippines_ in complex with 4E11 or 513 suggest that the designed substitutions Thr33Val, Ala55Glu in VH and Glu59Gln, Ser60Trp in VL improved affinity towards DENV4_Philippines_ (Fig. 10). Moreover, the A-strand of DENV4_Philippines_ (residues 305–310) has higher shape complementarity in complex with 513 and led to the formation of new H-bonds between Lys305 and Glu^L57^ and between Phe306 and Arg^L54^. Both these interactions are well conserved for over 95% of the equilibrated trajectory. Additional contacts between Glu327 with Gln^L59^ and Trp^L60^ are also conserved with over 75% H-bond occupancy (Table S2). In contrast, these favorable interactions are not observed in the case of 4E11 (Fig. 11), which has Asn^L57^, Glu^L59^ and Ser^L60^ respectively at those positions. Residue His390 also engages in a stabilizing strong H-bond interaction (95% occupancy) with Tyr^L32^ of 513, an interaction that is absent in the case of 4E11. These improved as well as additional contacts account for the net increase in the affinity of 513 for DENV4_Philippines_ strain compared to 4E11.

## Discussion

MD simulations suggest that the broad recognition by 513 of the four DENV serotypes results from a combination of a larger surface area buried upon complexation compared to 4E11 and specific interactions with conserved functional epitope core residues consisting of Lys310, a polymorphic site 323, Leu387 and Leu/Ile389. This is in agreement with previous experimental observations that were made for the interactions of mAb 4E11 with DENV1-4^32^. While hydrophobic interactions such as those mediated by Leu387 and Leu/Ile 389 remain similar between 4E11 and 513, the conserved charged residue Lys310, a residue deemed important for antibody-antigen recognition across all four serotypes, contributes ~1–2 kcal/mol more to the interactions with 513 (Fig. 6). Additionally, an interaction made by 513 with polymorphic residue 323 (Fig. 1A) leads to an additional ~1–9 kcal/mol for all four serotypes (Fig. 6).

Of the six mutations introduced in 4E11 to obtain 513 (CDR-L1: Arg31Lys, Thr33Val; CDR-L2: Asn57Glu, Glu59Gln, Ser60Trp; CDR-H2: Ala55Glu) (Fig. 5), it is clear that Arg (the original residue of 4E11) and Gln are largely preferred at positions 31 (CDR-L1) and 59 (CDR-L2) respectively. Mutations at Glu^L57^ and Trp^L60^ also lead to substantial increases in affinity for 513^33,34^. Glu^H55^ has a very large contribution in improving the interactions of 513 with the four serotypes. Indeed, computational Ala mutation analysis (Fig. 7) shows that residue at position H55 is the most important. It also has a large network score (Fig. 8) calculated from the orthogonal inter-residue interaction approach. In addition to the contacts observed in the crystal structure, MD simulations suggest that Glu^H55^ in 513 forms a stable interaction with the strictly conserved charged Lys310 in the DENV serotypes. The charge complementarity is further maintained in this region as the adjacent conserved acidic residue, Glu311, forms a stable interaction with Lys^L31^ in the 513 (CDR-L1) paratope. In addition, Glu^H55^ recognizes and stabilizes residue 323, which is a polymorphic residue on the DENV epitope, with significantly higher binding energy. In contrast to the observations made in the crystal structure (wherein Lys^H31^ is observed to form a H-bond with Lys323), MD simulations for all four DENV serotypes bound to 513 indicate that Asp^H31^ and Glu^H55^ stabilize this polymorphic site in concert. The Ala55Glu mutation increases charge complementarity across the paratope-epitope in this region, while the extended side chain of Glu endows broader specificity with increased contributing surface area. Importantly, the energetic contribution of Glu^H55^ was confirmed experimentally by site-directed mutagenesis followed by affinity measurement using ITC: Glu55Ala mutation in 513 showed similar affinity as the 4E11 WT, whereas Ala55Glu mutation in 4E11 showed enhanced affinity towards DENV2 DIII (Fig. S5 and Table 3).

The second major contributor towards the binding efficacy of 513 identified from our work is the Asn57Glu (CDR-L2) mutation, which specifically elicits favorable response due to charge complementarity for DENV2 and 4. The other two most important contributing residues are Tyr^H34^ and Tyr^L32^ (Figs 5 and 8). These two paratope residues, conjointly with Asp^H32^ and Lys^L31^, engage in stable H-bond interactions with the A-strand (311, 312), B-strand (323) and G strand (390) residues (Table S2) forming strong interactions at the periphery of the interface. Moreover, Gln and Trp residues at positions 59 and 60 (CDR-L2) form stable H-bonds with Glu327 specific for DENV4-513 complex that further enhances binding energetics. This bond is not observed in other DENV serotypes due to conformational variability.

In general, 513 is endowed with cross-neutralizing and enhancing activity for DENV1-4 serotypes, however significant differences in the binding efficacies were observed within DENV4 strains (see Table 2 of ref.^14^) such as DENV4_N.Cal_ and DENV4_Phillipines_. From our MD simulations, subtle sequence variations affect the local conformation of the functional epitope, altering shape complementarity and hence antibody binding. However, even though the binding constants are significantly different, the adjacent regions around the mutations largely preserve charge complementarity and the paratope-epitope interface.

In conclusion, a combination of physico-chemical and structural changes brought about by engineering six mutations in 4E11 result in the generation of mAb 513, which potently inhibits all four DENV serotypes. Combining MD simulations and residue-network analyses can therefore be a powerful complement to crystallographic and binding data and illuminate detailed interactions that underpin the differential binding of antibodies to homologous viral antigens. This has implications for the design of therapeutic cross-reactive antibodies against emerging viruses and also for the design of vaccines by “reverse vaccinology”.

## Materials and Methods

### Homology Modeling

The Kabat scheme^21^ was used, for defining and numbering the CDRs of 513, 4E5A and 4E11^15^ (http://www.bioinf.org.uk). To identify the interactions and hot spots of binding energies between 513 or 4E11 with various dengue serotypes, we built their respective homology models. The four dengue serotypes DENV1 (Hawaii/1944), DENV2 (Vietnam/2007), DENV3 (Nicaragua/2010), DENV4 (New Caledonia/2009) were modeled using template structures from 3UZQ^15^, 3UZV^15^, 3UZE^15^, 3UYP^15^ using the program Modeller^35^. Table S2 shows the list of PDB structures used to model the virus isolates as well as the mAbs and the complex structures. Point mutations in various virus serotypes were incorporated in the models and missing regions, if any, were modeled. The scFv513 and 4E11 antibody structures were extracted from the crystal structures 4UDZ and 3UZQ, followed by modeling the complexes with the respective serotypes. The files for electrostatic calculations were generated using PDB2PQR^36^ server and APBS^37^ plug-in in PyMol^38^ was used for electrostatic surface illustrations.

### Molecular Dynamics

The modeled complexes were subjected to molecular dynamics simulations for further refinement. The ff99SB force field as implemented in the program AMBER^39^ was used. In all the complexes, hydrogen atoms were added using the *Xleap* module of Amber12^39^ to prepare the systems for MD simulations. Each system was neutralized by adding sufficient number of Na^+^ and Cl^-^ counter ions. All the 8 complexes were then solvated in an octahedral box with TIP3P^40^ water molecules that extended 10 Å from any protein atom. The short range non-bonded van der Waals interactions were truncated at 9 Å while the long range electrostatics were approximated by the particle mesh Ewald^41^ method. The covalent bonds involving hydrogen atoms were constrained using SHAKE^42^. The *Sander* module was used for minimization of the complexes with 250 steps of steepest decent algorithm, followed by 8000 steps of conjugate gradient algorithm. Initially, the antibody atoms, solvent water molecules and counter ions were relaxed, by keeping the virus residues restrained. This was followed by unrestrained energy minimization to remove any steric clashes. The systems were subject to over 250 ps of heating from 50 to 300 K with weak restraints on the heavy atoms, followed by gradual reduction of the restraints over the next 250 ps, until the restraints were reduced to 0. For next 2 ns the system was equilibrated at 300 K under 1 atm constant pressure. After equilibration production runs of 50 ns with 2 fs time step were carried out on each complex and the atomic coordinates were saved every 10 ps. Analyses of the simulation trajectories were performed using the *ptraj* module in Amber. The conformations generated were oriented to a common frame to remove overall translation/rotations. The temporal evolution of the RMSD of the Cα atoms of various sampled conformations suggested that the systems had equilibrated within 10 ns. The free energy binding was calculated for each complex using MMPBSA^43,44^ methodology. For the binding energy calculations, a total of 50 structures were extracted at regular intervals from last 30 ns of the equilibrated trajectory. Water molecules and ions were stripped from all extracted snapshots and born implicit solvent model of 5 (igb = 5) was used to compute the free energies of binding. The effective binding energies were decomposed into contributions of individual residues using MMGBSA energy decomposition scheme. To investigate the impact of mutations in the antibodies on serotype recognition, selected hot-spot residues from the antibodies in each complex were computationally mutated to alanine. The free energy changes (*ΔΔG*_bind_) were defined as *ΔG*_bind_ (original residue) - *ΔG*_bind_ (alanine mutant) for the alanine mutation. A negative *ΔΔG*_bind_ value for that specific residue from the antibody indicated that the existing residue has a more favorable binding towards the respective DENV serotype. The solvent accessible surface areas were calculated for every 50^th^ frame in the equilibrated trajectory. Using the average values obtained above, BSA values were then calculated as the sum of the solvent accessible surface area of individual molecules minus the solvent accessible surface area of their complex. Calculations were performed using *Naccess* V2.1.1 program^45,46^ with a probe radius of 1.4 Å. The simulated trajectory was viewed in VMD^47^ and figures were generated using PyMol^38^.

### Protein refolding and purification

*E. coli* BL21 (DE3) Rosetta T1R cells, harboring scFv513 WT, scFv513 Glu55Ala, scFv4E11 WT and scFv4E11 Ala55Glu plasmids respectively, were cultivated at 37 °C to an OD_600nm_ ~ 1. The proteins were then overexpressed by the addition of 0.5 mM IPTG at 18 °C for 18 h. Cells were pelleted and stored at −80 °C. Thawed pellets were resuspended in 30 mL of lysis buffer (20 mM Na-HEPES, pH 7.5, 0.5 M NaCl, 10% (v/v) glycerol) and sonicated. The lysates were cleared by centrifugation at 58,000 g for 30 min. The pellets were resuspended in 50 mL of solubilizing buffer (20 mM Na-HEPES, pH 7.5, 0.5 M NaCl, 10% (v/v) glycerol, 8 M urea), dialysed overnight at 4 °C against 2 L of dialysis buffer devoid of urea (20 mM Na-HEPES, pH 7.5, 0.5 M NaCl, 10% (v/v) glycerol). Overnight dialysed samples were further buffer exchanged against a new batch of 2 L dialysis buffer for 8 h at 4 °C and dialysed against another new batch of 2 L dialysis buffer overnight. The dialyzed protein was concentrated to 5 mL and was further purified by size exclusion chromatography. The purified proteins were concentrated to 5 mg mL^-1^ in 20 mM Na-HEPES, pH 7.5, 0.15 M NaCl, 5% (v/v) glycerol. Aliquots were flash frozen in liquid nitrogen and stored at −80 °C.

### Isothermal titration calorimetry (ITC)

Measurements of scFv513 WT, scFv513 Glu55Ala, scFv4E11 WT and scFv4E11 Ala55Glu binding to DENV2 DIII domain were performed with a MicroCal PEAQ-ITC (Malvern Instruments Limited). Titrations were performed at 25 °C with DENV2 DIII domain at a concentration of 55 μM in the sample cell, consisting of a single initial injection of 0.5 μL, followed by 39 injections of 1 μL of scFV513 Glu55Ala at a concentration of 800 μM, scFv4E11 WT at a concentration of 500 μM and scFv4E11 Ala55Glu at a concentration of 500 μM. For scFv513 WT, the titrations consisted of a single initial injection of 0.5 μL, followed by 19 injections of 2 μL of scFv513 WT at a concentration of 450 μM into the sample cell containing DENV2 DIII at a concentration of 55 μM. Two consecutive injections were separated by 2.5 min to reach the baseline. Thermodynamic data were analyzed with a single-site fitting model using MicroCal PEAQ-ITC analysis software provided by the manufacturer.

## Electronic supplementary material

Supplementary information
