# Supplementary material for: Molecular basis for dengue virus broad cross-neutralization by humanized monoclonal antibody 513
Source: Sci Rep. 2018 May 31;8:8449. doi: 10.1038/s41598-018-26800-y (PMC5981469; doi:10.1038/s41598-018-26800-y)
Supplement: Supplementary file 1 — Supplementary information [file 41598_2018_26800_MOESM1_ESM.docx]

**Molecular basis for dengue virus broad cross-neutralization by humanized monoclonal antibody 513**

Yee Hwa Wong^1,5#^, Akshita Kumar^1,3,4#^, Chong Wai Liew^5^, Kannan Tharakaraman^2^, Kannan Srinivasaraghavan^4^, Ram Sasisekharan^2,3^, Chandra Verma^1,4*^, Julien Lescar^1,3,5*^

Supplemental data

**This file contains 2 Tables and 6 figures.**

**Table S1. List of X-ray crystal structures used as templates to model DENV in complex with 513 and 4E11 respectively.**

| **Modeled Structure** | **PDB ID** | **Resolution (Å)** |
| --- | --- | --- |
| **DENV1 (Hawaii/1944)** | **3UZQ** | **1.60** |
| **DENV2 (Vietnam/2007)** | **3UZV** | **2.10** |
| **DENV3 (Nicaragua/2010)** | **3UZE** | **2.04** |
| **DENV4 (New Caledonia/2009)** | **3UYP** | **2.00** |
| **scFv4E11** | **3UZQ** | **1.60** |
| **scFv513** | **5AAM** | **2.49** |

**Table S2. H-bonds identified from respective paratope residues in DENV1-4 serotypes in complex with 513 and 4E11.**

| **Virus** | **Denv-1 Hawaii** | | **Denv-2 Vietnam** | | **Denv-3 Nicaragua** | |
| --- | --- | --- | --- | --- | --- | --- |
|  | **: 513** | **: 4E11** | **: 513** | **: 4E11** | **: 513** | **: 4E11** |
| **305** |  |  | Lys (NZ): Glu^L57^(OE1/2) | Lys (NZ): Asn^L57^(OD1) | Thr (OG1): Arg^L54^ (NH1) | Thr(OG1): Arg^L54^ (NH1) |
| **306** | Phe (O): Arg^L54^ (NH1/2) | Phe (O): Arg^L54^(NH2) | Phe (O): Arg^L54^(NH1/2) | Phe (O): Arg^L54^(NH1/2) | Phe (O): Arg^L54^(NH1/2) | Phe (O): Arg^L54^(NH1/2) |
| **307** | Lys (NZ): Gln^L59^(OE1)  Lys (NZ): Glu^H102^(O) | Lys (NZ): Glu^L59^(OE1/2)  Lys (NZ): Glu^H102^(O) | Lys (NZ): Gln^L59^(OE1)  Lys (NZ): Glu^H102^(O) | Lys (NZ): Gln^L59^(OE1/2)  Lys (NZ): Glu^H102^(O) |  |  |
| **308** | Leu (N): Glu^H102^(OE2) | Leu(N): Glu^H102^(OE2)  Leu(N): Glu^H102^(O) | Val (N): Glu^H102^(OE2) | Val (N): Glu^H102^(OE2) | Lue (N): Glu^H102^(OE2) | Leu (N): Glu^H102^(OE2) |
| **309** | Glu (O): Tyr^H34^(N)  Glu (OE2): Arg^H99^(NH2)  Glu (OE2): Arg^H99^(NE) | Glu (OE1/2): Arg^H99^(NH2)  Glu (O): Thr^H31^(N)  Glu (OE2): Arg^H99^(NE) | Val (O): Tyr^H34^(N) |  | Lys (O): Tyr^H34^(N) | Lys (NZ): Asp^H32^(OD1/2)  Lys (NZ): Asp^H32^(O)  Lys (NZ): Thr^H33^(OG1) |
| **310** | Lys (NZ): Asp^H53^(OD1/2)  Lys (NZ): Glu^H55^(OE1/2) | Lys (NZ): Lys^H31^ (O)  Lys (NZ): Asp^H53^(OD1/2) | Lys (NZ): Lys^H31^ (O)  Lys (NZ): Asp^H53^(OD1/2)  Lys (NZ): Glu^H55^ (OE1/2) | Lys (NZ): Lys^H31^ (O)  Lys (NZ): Asp^H53^(OD1/2) | Lys (NZ): Asp^H53^(OD1)  Lys (NZ): Glu^H55^(OE2)  Lys (NZ): Lys^H31^(O) | Lys (NZ): Asp^H53^(OD1/2)  Lys (NZ): Lys^H31^(O) |
| **311** | Glu (N): Tyr^H34^(OH)  Glu (OE1/2: Lys^L31^(NZ) | Glu (OE1/2): Arg^L31^(NH1/2) | Glu (N): Tyr^H34^ (OH)  Glu (OE1/2): Lys^L31^(NZ) | Glu (OE2): Tyr^L32^(OH)  Glu (N): Tyr^H34^(OH) | Glu (N): Tyr^H34^(OH)  Glu (OE1/2): Lys^L31^(NZ) | Glu (OE1/2): Tyr^H34^(OH) |
| **312** | Val (N): Tyr^L32^(OH) | Val (N): Tyr^L32^(OH) | Ile (N): Tyr^L32^(OH) |  |  |  |
| **323** | Gln (NE2): Asp^H32^(O) | Gln (NE2): Asp^H32^(O)  Gln (N): Tyr^H34^(OH) | Arg (NH1/2): Glu^H55^(OE1/2)  Arg (NH1/2): Asp^H32^(OD1/2)  Arg (NH2): Lys^H31^ (O) |  | Lys (NZ): Asp^H32^(OD1/2)  Lys (NZ): Glu^H55^(OE1/2) |  |
| **325** | Lys (NZ): Tyr^H106^(OH) |  | Gln (OE1): Arg^H99^(NH2)  Gln (NE2): Arg^H99^(NH2) |  | Glu (OE1/2): Arg^H99^(NH1/2) | Glu (OE1/2: Arg^H99^(NH1/2)  Glu (OE1/2): Lys^H3^(NZ) |
| **327** |  |  |  |  |  | Lys (NH1): Glu^L59^(OE1)  Lys (NZ): Glu^L59^(OE1/2)  Lys (NZ): Ser^L56^(OG)  Lys (NZ): Tyr ^L53^(OH) |
| **361** |  |  |  |  |  | Lys (O): Lys^H3^(NZ) |
| **362** | Glu (O): Arg^H99^(NH1/2)  Glu (O): Arg^H99^(NH1) | Glu (O): Arg^H99^(NH1/2) | Asp (O): Arg^H99^(NH2)  Asp (OD1/2): Arg^H99^(NH1) | Asp (OD1/2): Lys^H3^(NZ) | Glu (O): Arg^H99^(NH2) |  |
| **366** |  |  |  |  |  |  |
| **388** | Lys (O): Asn^L34^(ND2) | Lys (O): Asn^L34^(ND2) |  |  | Lys (NZ): Asn^L34^(OD1)  Lys (O): Asn^L34^(ND2) |  |
| **390** | Ser (N): Tyr^L32^(O)Å | Ser (N): Tyr^L32^(O) | Asn (N): Tyr^L32^(O) | Asn (N): Tyr^L32^(O) | Asn (N):Tyr^L32^(O)  Asn (N):Tyr^L32^(OH) | Asn (N): Tyr^L32^(O) |

| **Virus** | **Denv-4 New Caledonia** | | **Denv-4 Philippines** | |
| --- | --- | --- | --- | --- |
|  | **: 513** | **: 4E11** | **: 513** | **: 4E11** |
| **305** | Lys (NZ): Glu^L57^(OE1/2) | Lys (NZ): Asn^L57^(OD1) | Lys (NZ): Glu^L57^(OE1/2) |  |
| **306** | Phe (O): Arg^L54^(NH1/2) | Phe(O): Arg^L54^(NH2) | Phe(O): Arg^L54^(NH2) |  |
| **307** | Ser (OG): Glu^H102^(OE1) |  | Ser (N): Glu^H102^(O) | Ser (OG): Glu^H102^(OE1)  Ser (O): Glu^H102^(N) |
| **308** | Ile (N): Glu^H102^(OE1) | Ile (N): Glu^H102^(OE2) | Ile (N): (Glu^H102^(OE1) | Ile(N): Glu^H102^(OE1) |
| **309** | Asp (OD1): Arg^H99^(NH2) | Asp (OD1): Arg^H99^(NH2)  Asp (OD2): Arg^H99^(NE) | Asp (OD1): Arg^H99^(NH2)  Asp (OD1): Arg^H99^(NE) | Asp (OD2): Thr^H33^(OG1)  Asp (OD2): Arg^H99^(NH2)  Asp (OD2): Arg^H99^(NE) |
| **310** | Lys (NZ): Asp^H53^(OD1/2)  Lys (NZ): Lys^H31^(O)  Lys (NZ): Glu^H55^(OE1/2) | Lys (NZ): Asp^H53^(OD1/2)  Lys (NZ): Lys^H31^(OD2) | Lys (NZ): Asp^H53^(OD1/2)  Lys (NZ): Glu^H55^(OD2)  Lys (NZ): Val^H33^(O) | Lys (NZ): Asp^H53^(OD1/2) |
| **311** | Glu (OE1/2): Lys^L31^(NZ) | Glu (OE1.2): Arg^L31^(NH1/2) |  | Glu(N): Tyr^H32^(OH) |
| **312** | Met (N): Tyr^L32^(OH) | Met(N): Tyr^L32^(OH) |  |  |
| **323** | Lys (NZ): Glu^H55^(OE1/2)  Lys (NZ): Asp^H32^(OD2)  Lys (NZ): Lys^H31^(O) |  | Lys (NZ): Asp^H32^(O)  Lys (NZ): Asp^H32^(OD1/2)  Lys (NZ): Glu^H55^(OE2) | Lys (NZ): Asp^H32^(O)  Lys (NZ): Asp^H32^(OD2) |
| **325** |  |  | Lys (NZ): Glu^H102^(O) |  |
| **327** | Glu (OE1): Gln^L59^(NE2)  Glu (OE2): Trp^L60^(N) |  | Glu (OE1/2): Gln^L59^(NE2)  Glu (OE2): Trp^L60^(N)  Glu (OE2): Tyr^L53^(OH) |  |
| **361** | Thr (OD1): Gln^H3^ (NE1/2) | Thr (O): Tyr^H106^(OH) |  |  |
| **362** | Asn (O): Arg^H98^(NH1/2)  Asn (OD1): Arg^H98^(NH1)  Asn (ND2): Gln^H3^(NE2) | Asn (O): Arg^H98^(NH1)  Asn (OD1): Arg^H98^(NH1) |  | Asn (OD1): Tyr^H106^(OH)  Asn (ND2): Tyr^H106^(OH) |
| **366** |  |  |  |  |
| **388** |  | Thr (O): Asn^L34^(ND2 ) |  | Thr (N): Asn^L34^(OD1)  Thr (O): Tyr ^L32^ (OH) |
| **390** | His (N): Tyr^L32^(O) | His (N): Tyr^L32^(O) | His (N): Tyr^L32^(O) |  |

The residue number for 513 and 4E11 is mentioned using Kabat convention.

During ptraj calculations the acceptor –donor distance cut off was less than 3.5 Å and acceptor –H-bond angle was greater than 120**°**.

The % occupancy of H-bond was greater than 50% in all the cases.


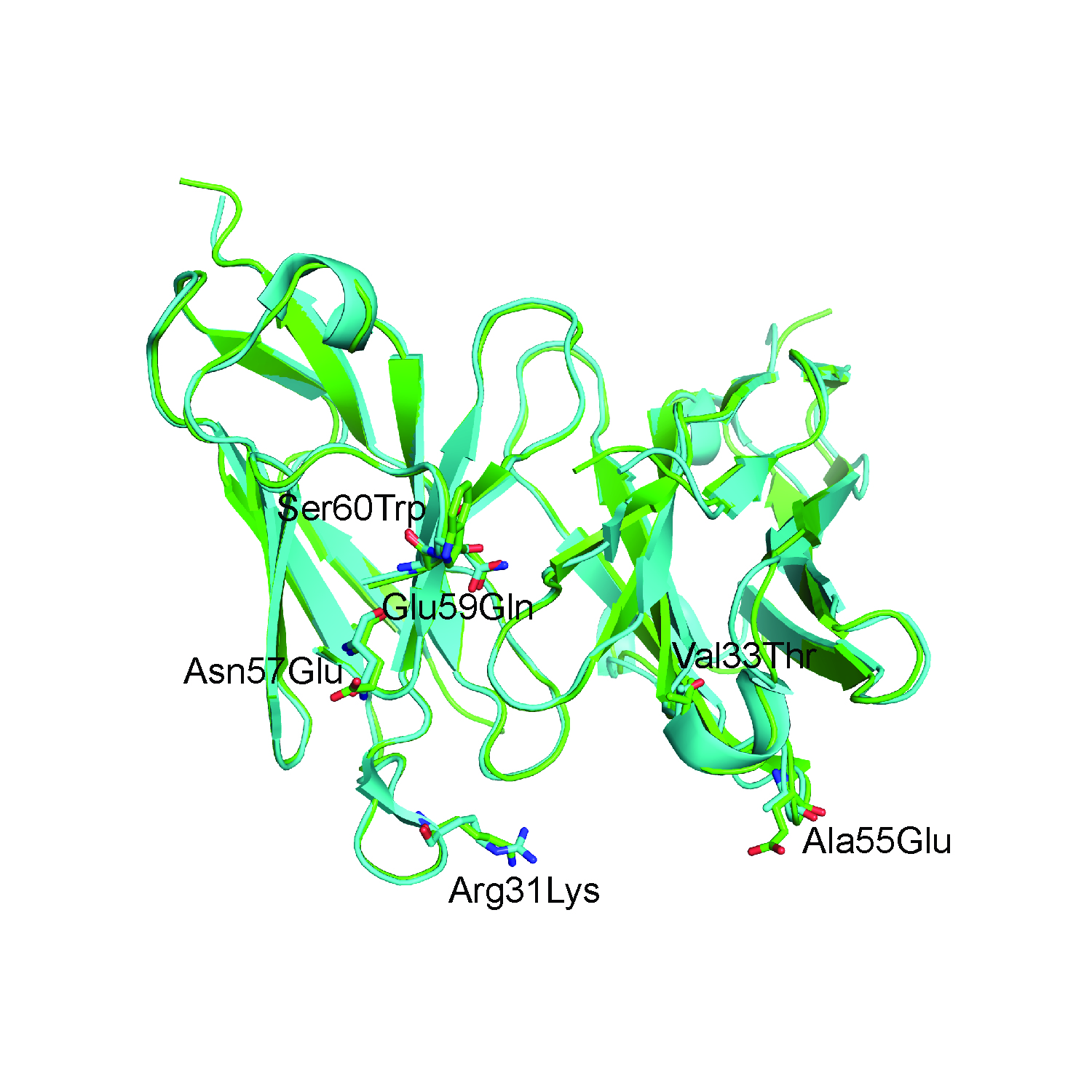


**Figure S1.** Superposition of the structures of scFv513 (green) and scFv4E11 (blue) with the 6 residues mutated between them displayed as stick and labeled.


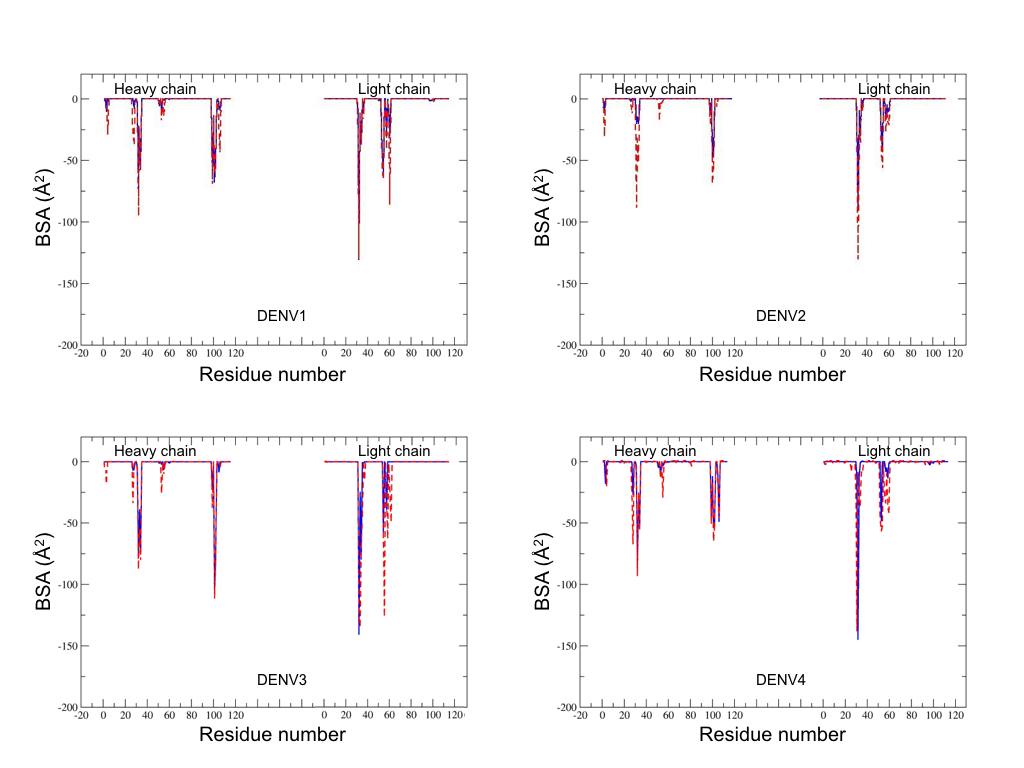


**Figure S2**. BSA values for 513 (red) vs. 4E11 (blue) heavy chain and light chain when bound to the respective DENV serotypes.


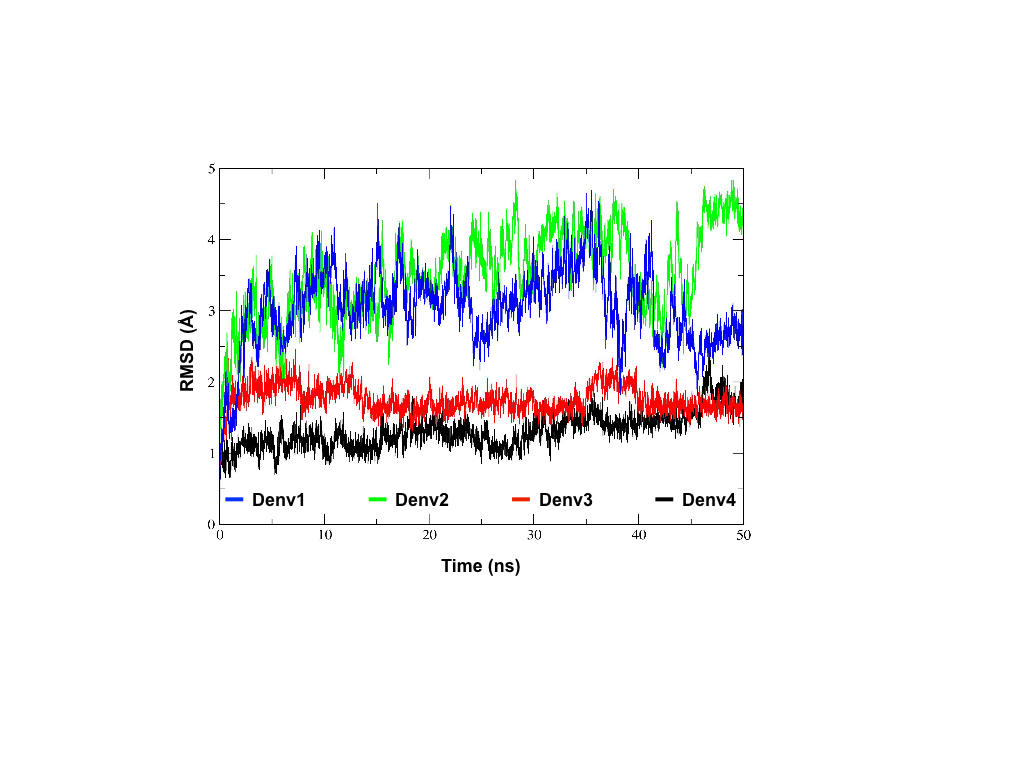


**Figure S3.** RMSD plot for DENV serotypes in their free state for a 50 ns MD trajectory. Denv1 (blue), Denv2 (green), Denv3(red), Denv4 (black).


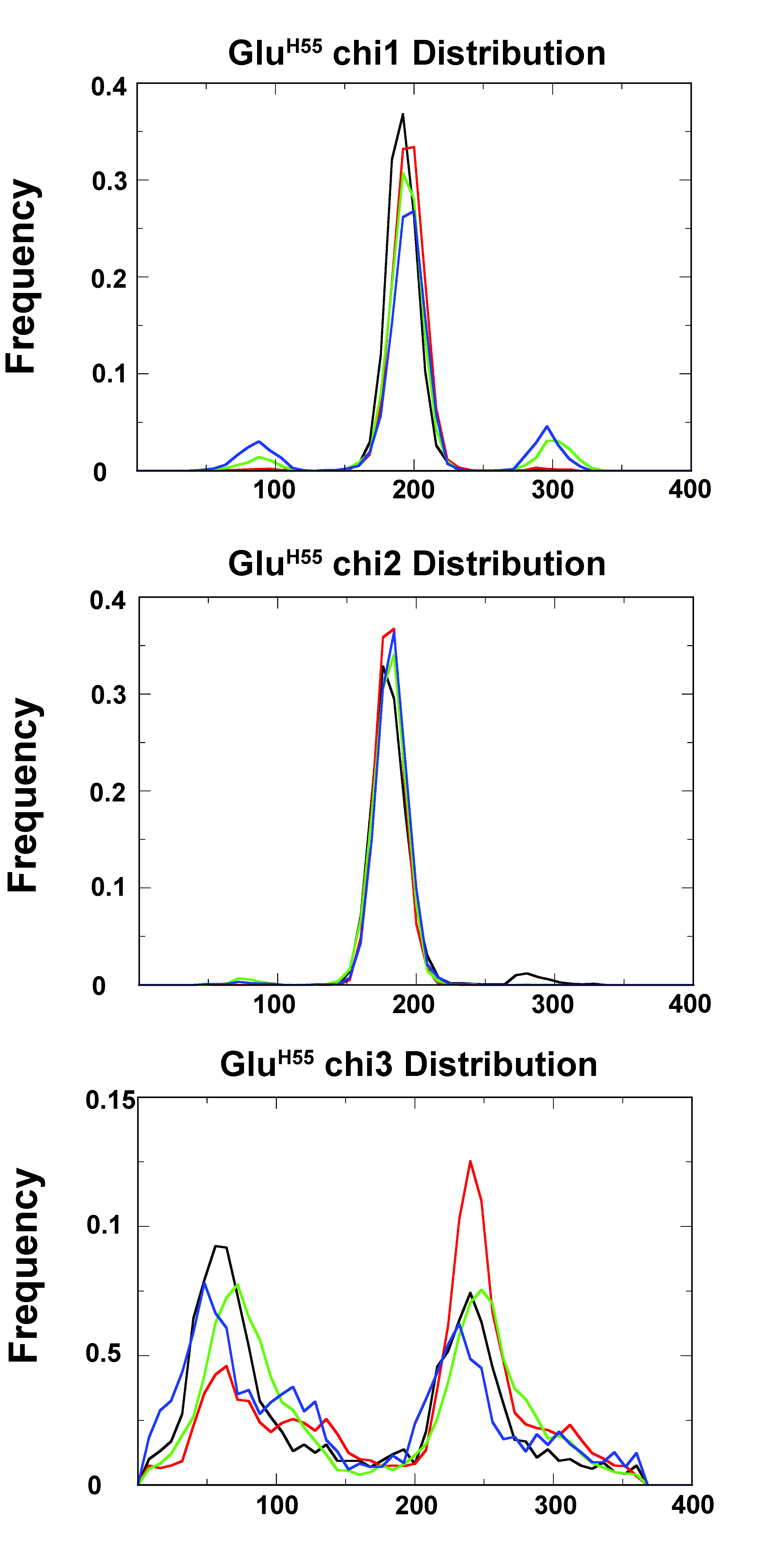


**Figure S4.** Glu^H55^ Chi1, Chi2 and Chi3 distribution plots for the equilibrated MD trajectory depicting no major structural changes upon interaction with DENV1 (black), Denv2 (red), Denv3 (green), Denv4(blue) respectively.


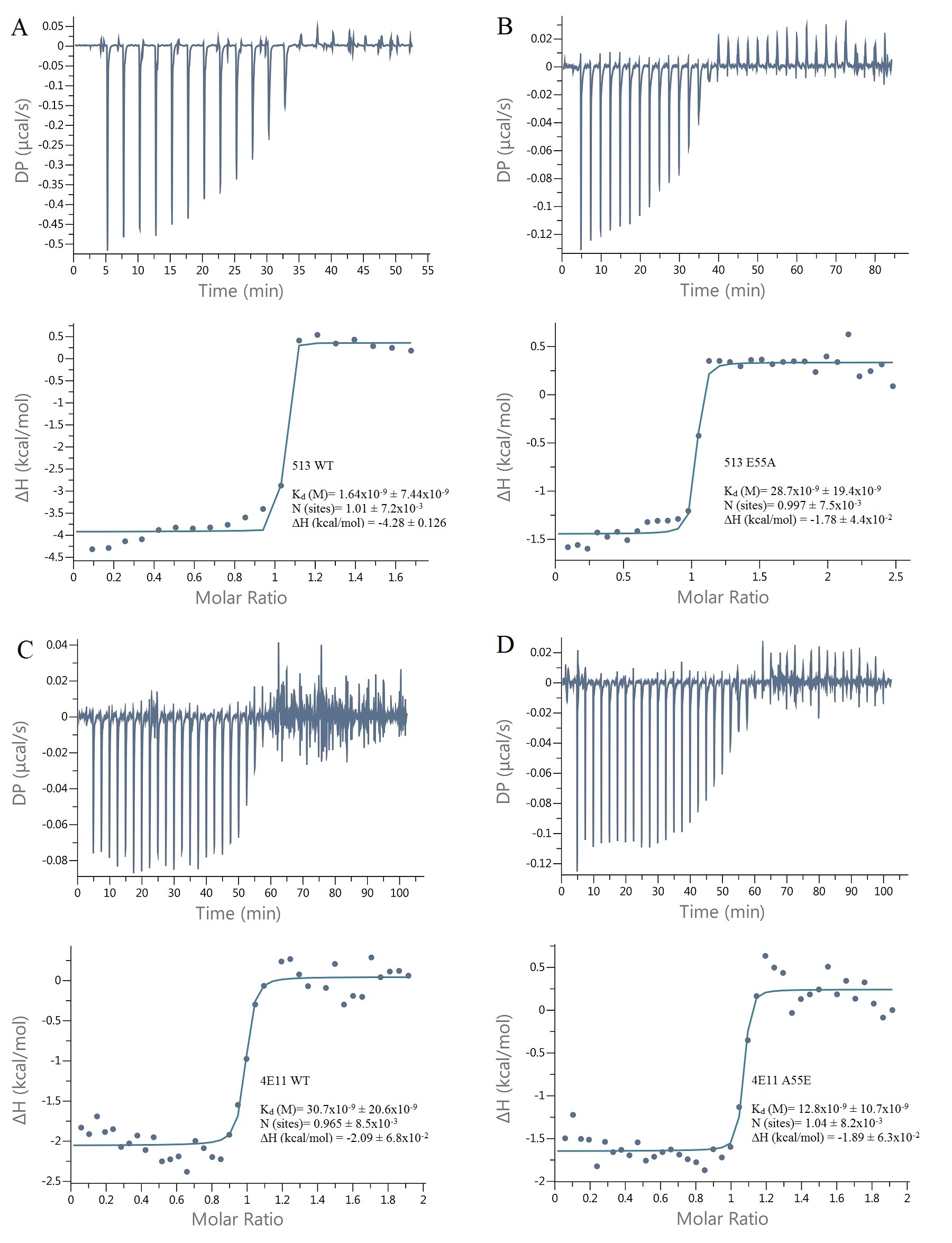


**Figure S5. ITC analysis of binding thermodynamics at position H55.** The binding affinity of **A.** scFv513 WT, **B.** scFv513 Glu55Ala mutant, **C.** scFv4E11 WT, **D.** scFV4E11 Ala55Glu mutant binding to DENV2 DIII. See text for details.


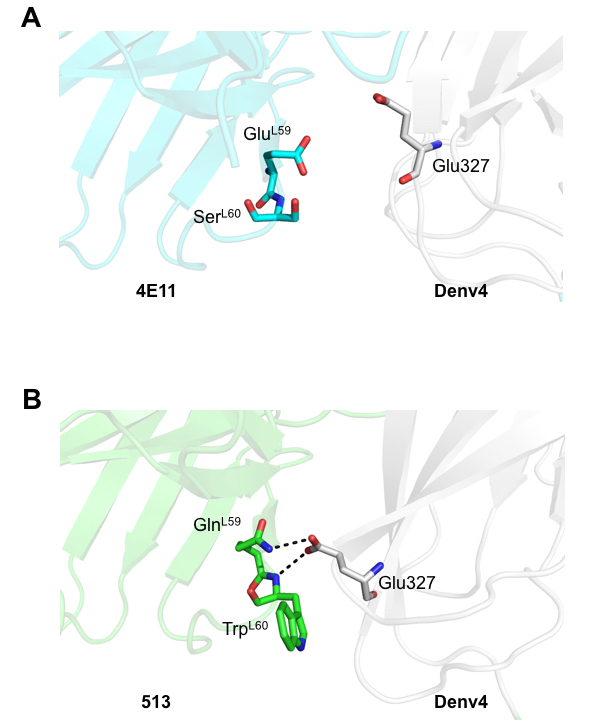


**Figure S6.** Interaction of residue Glu327 in DENV4 serotype with (A) 4E11 and (B) 513 antibody.
